# Dual Role of Vitamin C-Encapsulated Liposomal Berberine in Effective Colon Anticancer Immunotherapy

**DOI:** 10.3390/ph17010005

**Published:** 2023-12-20

**Authors:** Martyna Mianowska, Magdalena Zaremba-Czogalla, Adrianna Zygmunt, Mohamed Mahmud, Regine Süss, Jerzy Gubernator

**Affiliations:** 1 Department of Lipids and Liposomes, Faculty of Biotechnology, University of Wroclaw, 50-383 Wroclaw, Poland; magdalena.zaremba-czogalla@uwr.edu.pl (M.Z.-C.); adrianna.zygmunt@uwr.edu.pl (A.Z.); mohamedzwawi@yahoo.com (M.M.); 2 Department of Medical Genetics, Faculty of Health Sciences, University of Misurata, Misurata 2478, Libya; 3 Institute of Pharmaceutical Sciences, Department of Pharmaceutics, Albert Ludwig University of Freiburg, Sonnenstr. 5, 79104 Freiburg, Germany; regine.suess@pharmazie.uni-freiburg.de

**Keywords:** colon cancer, CD47, berberine, liposomes, calreticulin, ICD

## Abstract

The aim of the study was to achieve effective colon anticancer immunotherapy using the alkaloid berberine. In the presented paper we attempt to develop a formulation of berberine loaded into liposomal carriers using the vitamin C gradient method, characterized by efficient drug encapsulation, high stability during long-term storage, low drug release in human plasma with specific cytotoxicity towards colon cancer cells. Liposomal berberine was responsible for the induction of oxidative stress, the presence of Ca^2+^ ions in the cytosol, the reduction of Δψm, and ATP depletion with a simultaneous lack of caspase activity. Moreover, treatment with liposomal berberine led to CRT exposure on the surface of cancer cells, extracellular ATP, and HMGB1 release. The above-described mechanism of action was most likely associated with ICD induction, contributing to the increased number of phagocytic cancer cells. We have shown that cancer cells treated with liposomal berberine were phagocytosed more frequently by macrophages compared to the untreated cancer cells. What is more, we have shown that macrophage pre-treatment with liposomal berberine led to a 3-fold change in the number of phagocytosed SW620 cancer cells. The obtained results provide new insights into the role of berberine in maintaining the immune response against colorectal cancer.

## 1. Introduction

Every year, cancer causes millions of deaths worldwide. The third most frequently diagnosed and fourth deadliest malignancy for both males and females is colorectal cancer (CRC) [1]. Phases of colorectal cancer development include occupation of different layers of the colon wall: initially as a polyp of the mucosa (or as a benign lesion called an adenoma, which can develop into a malignant lesion), followed by the submucosa, thick muscle layers, and finally the serous membranes (subserosa and serosa) [2].

Risk factors that may increase the chance of an individual developing colorectal cancer include lifestyle factors such as poor dietary habits (high consumption of processed/red meat without fruits and vegetables, meals high in fat and proteins while low in fiber), obesity, physical inactivity, type 2 diabetes mellitus, cigarettes, and alcohol consumption) and genetic factors (familial adenomatous polyposis and hereditary non-polyposis colorectal cancer, Lynch syndrome and chronic colitis due to inflammatory bowel disease (IBD)) [3,4]. The most frequently observed symptoms of colorectal cancer include abdominal pain, changes in bowel habits, rectal bleeding, and anemia [3].

Based on the demographic assessment, an increase in the number of cases is expected, which in 2030 will reach over 2.2 million new cases and 1.1 million deaths [5,6,7].

In 2011, D. Hanahan and R.A. Weinberg described 10 cancer hallmarks, which include immunoevasion and resistance to antitumor immunity [5,6]. The immune system is responsible for finding and eliminating potentially dangerous cells (including neoplastically transformed ones), and tumor development is probably a result of immune response failure [7]. The elimination of cancer cells follows the recognition of tumor antigens by the immune system. These antigens are usually divided into two main groups: tumor-specific antigens (TSAs)—molecules that are unique to cancer cells; and tumor-associated antigens (TAAs)—molecules that are expressed differently by transformed cells and normal cells [7,8,9]. It is believed that overexpression of TAAs (which play a key role as checkpoints for the immune cells during verification of whether a given cell is self or foreign (non-self)) is mainly responsible for the low immunogenicity of cancer cells [10]. One of the discovered complexes enabling the escape of cancer cells from macrophage-mediated phagocytosis is the CD47–SIRPα axis [11,12]. Based on these findings, it was assumed that inhibition of the CD47 receptor could serve as immune therapy for the restoration of immune surveillance [11,13].

Immunogenic cell death (ICD), in addition to induction-programmed cell death, stimulates the immune system to remove dead cancer cells from the patient’s body. The hallmarks of ICD include calreticulin (CRT) exposure on the cell surface and the release of ATP and HMGB1 protein into the extracellular space. Blocking of the CD47 receptor enables the recognition of cancer cells by binding the CRT present on their surface to the LRP-1/CD91 receptor occurring on the cell surface of macrophages [14,15,16]. The findings that certain drugs are able to induce the awakening of the immune response by releasing damage-associated molecular patterns (DAMPs) and generating ICD triggered investigations to look for these types of agents [17,18]. However, the application of traditional inducers of ICD in anti-cancer immunotherapy has been limited due to low levels of ICD induction. An explanation could be the fact that the reactive oxygen species (ROS) level generated in the endoplasmic reticulum (ER) lumen is not sufficient to induce oxidative stress leading to ICD [19]. In 2019, two research groups presented data demonstrating that the alteration of mitochondria in cancer cells could be an important target for the development of an efficient ICD inducer for use in cancer immunotherapy [19,20]. In cancer cells, alterations in different levels of oxidative phosphorylation (OXPHOS), especially inhibition or uncoupling of respiration or inhibition in the transport of ADP/ATP, are events that produce ATP depletion with the induction of selective cancer cell death. Many alkaloids selectively affect some functions of cancer cells’ mitochondria, including regulation of the respiratory chain and ATP production, increasing the ROS level, and decreasing mitochondrial membrane potential (Δψm) due to microdomains connecting the ER with the mitochondria [21].

**Figure 1:**
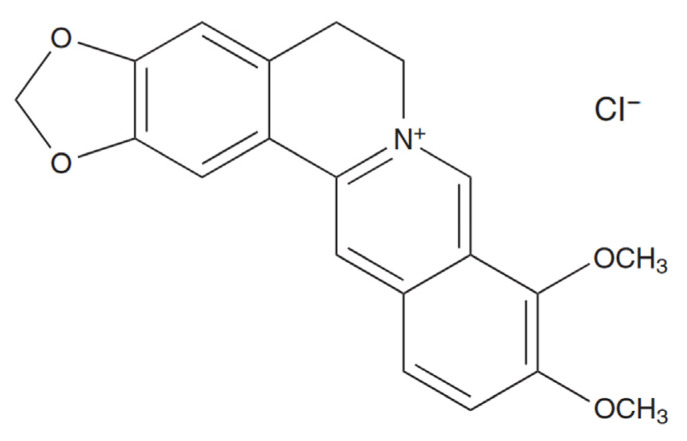
Chemical structure of berberine chloride [22].

**Figure 2:**
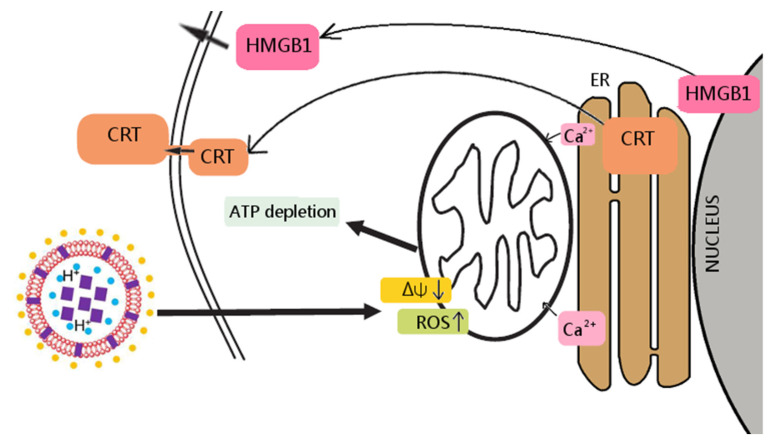
Berberine’s mechanism of action towards colon cancer cells. In the liposome scheme, berberine was marked as purple squares, vitamin C as blue circles, DSPE PEG-2000 as yellow circles.

The present study is focused on determining whether berberine, an isoquinoline quaternary alkaloid (Figure 1) [22], is able to induce selective ICD in colon cancer cells in vitro while conserving the principal characteristics of CD47-mediated cell death [17]. Although the direct anticancer effect of berberine has been well documented, its influence on the function of macrophages, in particular their antitumor activity, is largely unknown [23]. In this study, we demonstrated the anti-tumor effect of berberine along with its capacity to activate macrophages to be highly cytostatic against tumor cells in vitro (Figure 2).

According to the investigations, berberine has limited aqueous solubility (pH and temperature dependency). A slight increase in temperature results in an increase in its solubility—at 25 °C, berberine reaches a concentration of 5.27 mM, and at 37 °C, 8.50 mM [24,25]. Other factors affecting the pharmacokinetic profile of berberine include poor absorption from the digestive system, rapid metabolism through hepatic enzymes and interaction with P-glycoprotein [26,27,28], slow pharmacological action, and low stability (<5%) [26,27,28,29]. This limits the application of berberine as an ICD inducer, which could be used together with classic drugs or other factors that enhance the immune response. To overcome these limitations, we encapsulated berberine inside the unilamellar PEGylated liposomes with different pH gradients (including the vitamin C pH gradient method, which was invented in our laboratory). The vitamin C pH gradient allows for the encapsulation of a small amount of weak amphiphilic amines, increases activity as well as specificity, and simultaneously decreases toxicity toward normal cells [30,31,32]. Vitamin C increases the free radical production in cancer cells by promoting the Fenton reaction. The resulting liposomes are characterized by a high drug-to-lipid ratio, a long half-life of the encapsulated drug, prolonged circulation time, and the phenomenon of the EPR effect. We assessed the active loading method of encapsulation for berberine. At a neutral pH, a weak base exists in both non-ionized and ionized form, which can freely penetrate and accumulate inside the liposomes. Increasing the pH of the external buffer contributes to the shift of the equilibrium towards the non-ionized form, which leads to an increase in the amount of the non-ionized form of the weak base passing through the membrane and accumulating inside the liposomes [33]. The vitamin-C-loaded berberine formulation was found to be the best in terms of encapsulation efficiency and stability. Moreover, it showed the highest cytotoxicity against cancer cells while having a marginal effect on the viability of normal colon cells. The observed specific toxicity towards malignant cells could be connected with one of vitamin C’s mechanisms of action—the delivery of H_2_O_2_ to cancer cells, leading to cancer cell death [34]. 

## 2. Results

### 2.1. Characterization of Prepared Blank Liposomes

**Figure 3:**
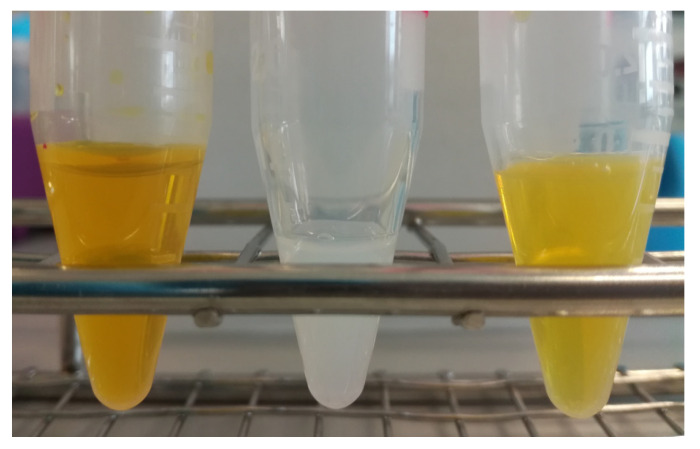
Preparation of liposomal berberine: (**left**) aqueous berberine solution, (**middle**) blank liposomes, (**right**) berberine-loaded liposomes.

The mean diameter size and PDI of the resulting formulations were determined and are summarized in Table 1. The sizes of berberine-loaded liposomes were in the range from 99.3 to around 118 nm, with a narrow size distribution (PDI) ranging from 0.032 to 0.052 for all formulations. The appearance of berberine-loaded liposomes had a yellow color without any crystal, as shown in Figure 3.

### 2.2. Optimization of the Berberine Active Loading inside the Liposomes

**Figure 4:**
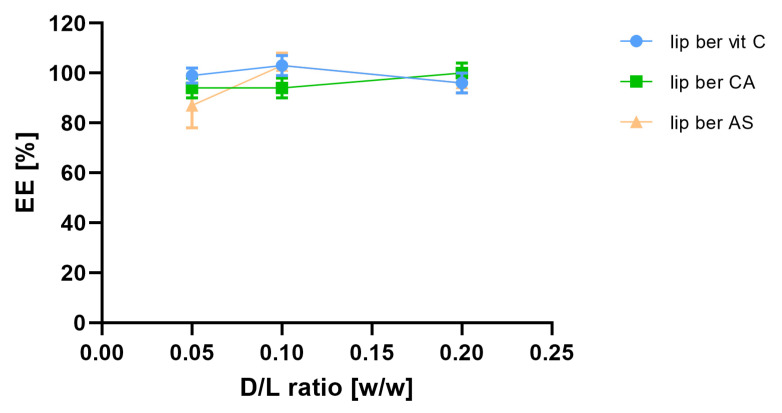
Influence of different berberine-to-lipid ratios on berberine EE%. Abbreviations: lip ber vit C—berberine-loaded liposomes with vitamin C, lip ber CA—berberine-loaded liposomes with citric acid, lip ber AS—berberine-loaded liposomes with ammonium sulfate. The results are shown as the mean ± SD of three replicates.

**Figure 5:**
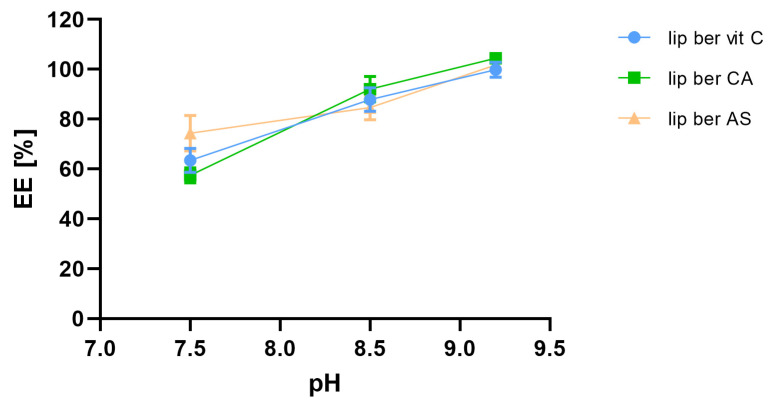
Influence of external pH on berberine encapsulation efficacy [EE%]. Abbreviations: lip ber vit C—berberine-loaded liposomes with vitamin C, lip ber CA—berberine-loaded liposomes with citric acid, lip ber AS—berberine-loaded liposomes with ammonium sulfate. The results are shown as the mean ± SD of three replicates.

**Figure 6:**
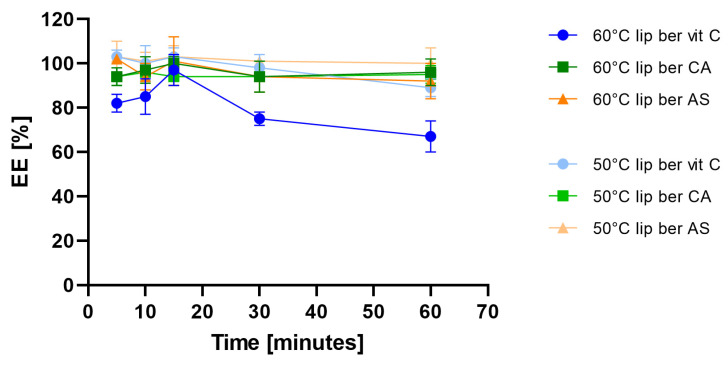
Influence of different temperatures on berberine encapsulation efficacy [EE%]. Abbreviations: lip ber vit C—berberine-loaded liposomes with vitamin C, lip ber CA—berberine-loaded liposomes with citric acid, lip ber AS—berberine-loaded liposomes with ammonium sulfate. The results are shown as the mean ± SD of three replicates.

In order to select optimal conditions for encapsulation of berberine inside liposomes, different drug-to-lipid molar ratios (0.05–0.2), different external buffer pHs (pH 7.5–9.2), different temperatures (50 °C and 60 °C), and time intervals (5–60 min) for different salt gradients were tested. High (>90%) encapsulation efficiency (EE%) could be achieved for a berberine-to-lipid molar ratio of up to 0.1, conducted at 50 °C for 15 min with external carbonate buffer (pH 9.2). The results are summarized in Figure 4, Figure 5 and Figure 6.

### 2.3. Cryo-TEM

**Figure 7:**
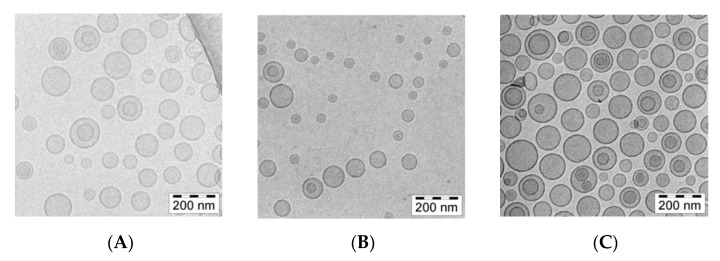
cryo-TEM micrographs of HSPC/Chol/DSPE-PEG_2000_ (5.5:4:0.5 mol/mol) liposomes loaded with berberine using different pH gradient agents at a 1:10 (*w*/*w*) drug-to-lipid ratio. The internal buffer was 300 mM ascorbic acid (**A**), 300 mM citric acid (**B**), or 300 mM ammonium sulfate (**C**). The entire bar represents 200 nm.

The cryogenic transmission electron (cryo-TEM) technique was applied to determine and visualize the physical state of the berberine inside lipid vesicles. This state can directly influence the dissolution rate of the drug from the liposomes and, thus, its anticancer activity [35]. Berberine does not form a low-soluble precipitate in the form of elongated bundles. In all formulations, fine grainy structures of berberine, hardly visible on the cryo-TEM images, are observed inside of liposomes, with some additional rod-shaped structures seen in certain citrate liposomes. All the data are summarized in Figure 7.

**Figure 8:**
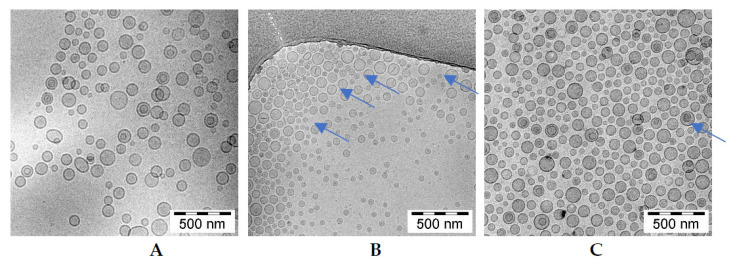
cryo-TEM micrographs of HSPC/Chol/DSPE-PEG_2000_ (5.5:4:0.5 mol/mol) liposomes loaded with berberine using different pH gradient agents at a 1:10 (*w*/*w*) drug-to-lipid ratio. The internal buffer was 300 mM ascorbic acid (**A**), 300 mM citric acid (**B**), or 300 mM ammonium sulfate (**C**). The entire bar represents 500 nm. The arrows indicate thin rod-like structure berberine inside liposomes.

A detailed analysis of the images presented in Figure 8 indicates only fine, grainy structures of the berberine inside ammonium sulfate liposomes. Similar fine grainy structures and occasional rod-shaped precipitates are seen in Citric acid-berberine formulation. The circular liposome shape remains unchanged. The sizes of liposomal formulations were in the range of 90 to around 120 nm, with PDI ranging from 0.03 to 0.12, and the lipid concentration was 10 mg/mL for all formulations.

### 2.4. Long-Term Stability of Liposomal Berberine

**Figure 9:**
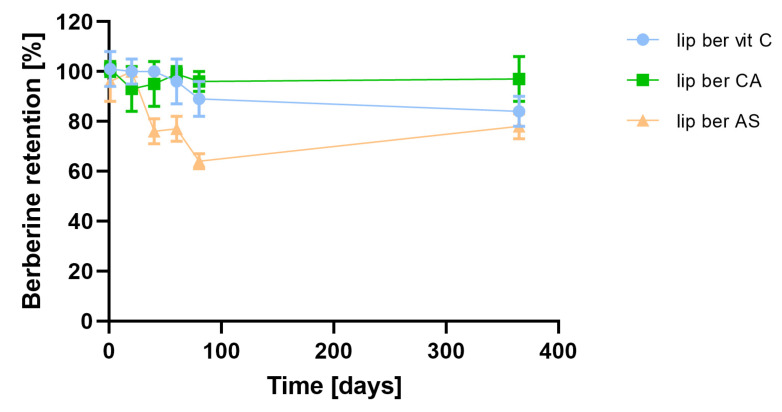
Influence of time on berberine retention. Abbreviations: lip ber vit C—berberine-loaded liposomes with vitamin C, lip ber CA—berberine-loaded liposomes with citric acid, lip ber AS—berberine-loaded liposomes with ammonium sulfate. The results are shown as the mean ± SD of three replicates.

In order to assess the preliminarily long-term stability of liposomal berberine, prepared liposomes were stored for 12 months at 4 °C. At selected time points, aliquots were withdrawn, and berberine retention was measured. Figure 9 shows that all selected formulations of berberine-loaded liposomes had desirable stability, although there was a slightly decreased berberine retention (16% vs. 22%) over the time period for two liposomal formulations.

### 2.5. Stability of Berberine-Loaded Liposomes in Presence of Human Plasma In Vitro

**Figure 10:**
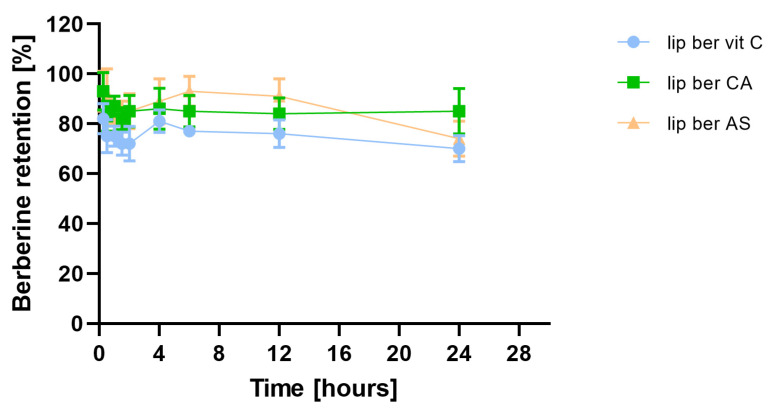
Influence of human plasma proteins on berberine retention. Abbreviations: lip ber vit C—berberine-loaded liposomes with vitamin C, lip ber CA—berberine-loaded liposomes with citric acid, lip ber AS—berberine-loaded liposomes with ammonium sulfate. The results are shown as the mean ± SD of three replicates.

Incubation of berberine-loaded liposomes with human plasma showed time-dependent, gradual berberine retention. This was minimal in the case of the berberine liposomal formulation with citric acid, which was found to exhibit higher stability in human plasma among all formulations. There was a 15% release of berberine from the formulation containing citric acid after 24 h of incubation, while ascorbic acid and ammonium sulfate formulations showed releases of 30 and 26% berberine, respectively (Figure 10).

### 2.6. Selective Cytotoxicity towards Colon Cancer Cells

The anti-proliferative effects of liposomal berberine on human colon cancer cell lines (LS180 and SW620) and the non-transformed colon fibroblast cell line CCD112CoN were assessed by an SRB assay. Cells were treated with serial dilutions of free berberine, liposomal berberine, and blank liposomes for 24, 48, and 72 h. Both cancer cell lines were sensitive to berberine in a dose- and time-dependent manner (see Supplementary Materials, Figures S1–S8). In addition, it can be perceived that loss of CCD112CoN viability was relatively not detected even at the highest concentration tested. Based on the survival curves for all formulations, a liposomal formulation containing berberine encapsulated via vitamin C gradient was selected for further study. The IC_50_ values were determined and summarized in Table 2.

### 2.7. Intracellular ATP Depletion

**Figure 11:**
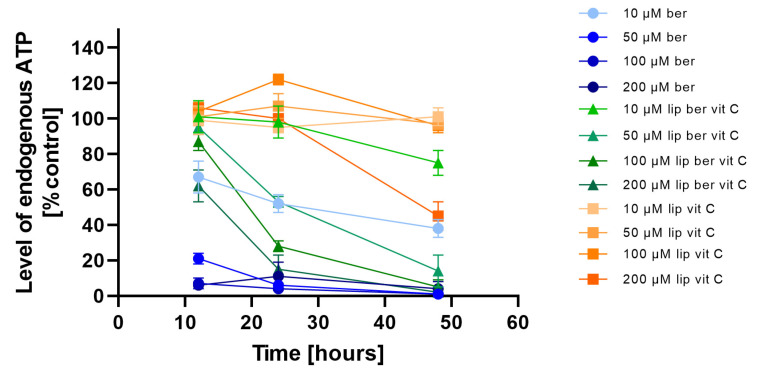
Intracellular ATP level in the LS180 cell line after administration of berberine-loaded liposomes, blank liposomes, and free berberine. RLU of untreated cells (control) was considered to be 100% of endogenous ATP level. Data represent the mean ± SD of three independent biological replicates.

**Figure 12:**
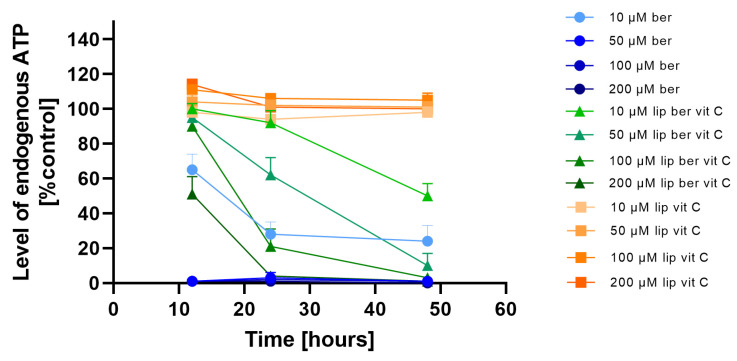
Intracellular ATP level in the SW620 cell line after treatment with berberine-loaded liposomes, blank liposomes, and free berberine. RLU of untreated cells (control) was considered to be 100% of endogenous ATP level. Data represent the mean ± SD of three independent biological replicates.

To investigate whether the observed decrease in cell viability is correlated with the induction of mitochondrial respiration dysfunction and decreased ATP production efficiency, the CellTiter-Glo 2.0 Cell Viability Assay was performed. We observed fast ATP depletion in both colon cancer cell lines after berberine and berberine-loaded liposomes (Figure 11 and Figure 12).

Subsequent experiments using the Caspase-Glo 3/7 assay, the Caspase-Glo 8 assay, and the Caspase-Glo 9 assay were performed to examine the type of cell death. When apoptosis is induced by a certain treatment, increased cleavage of caspases can be detected by an increase in luminescent signals compared to nontreated controls [36]. We observed that neither berberine nor berberine loaded liposomes with vitamin C-induced caspase-independent cell death in SW620 cancer cell lines (see Supplementary Materials, Figures S9–S14).

### 2.8. Berberine Induced Colon Cancer Cell Death via Oncosis

**Figure 13:**
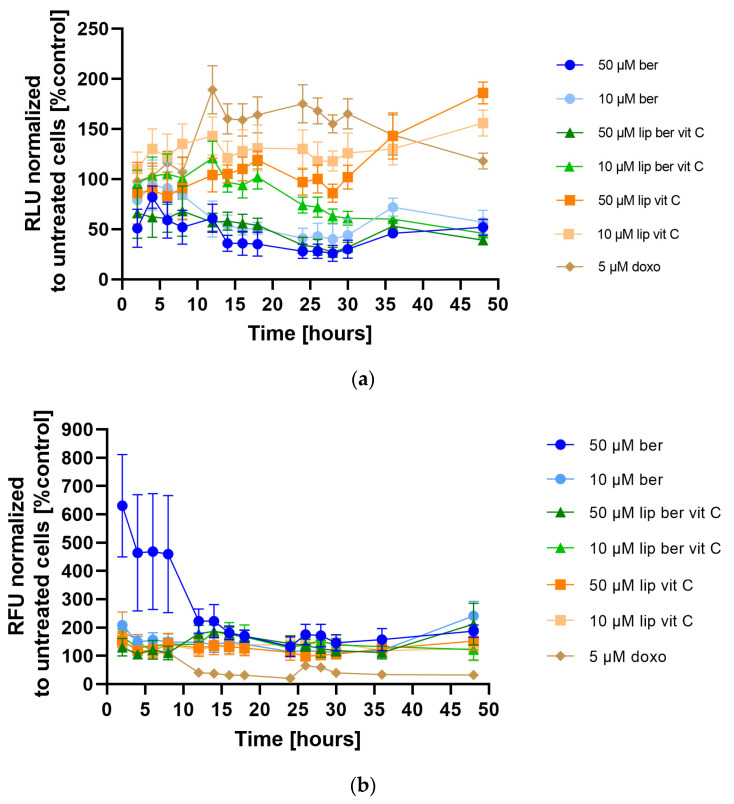
(**a**) Apoptosis and (**b**) Necrosis detection in the SW620 cell line after treatment with berberine-loaded liposomes, blank liposomes, and free berberine. (**a**) RLU and (**b**) RFU of untreated cells (control) was considered to be 100%. Data represent the mean ± SD of four independent biological replicates.

The absence of caspase activation and rapid ATP depletion observed after berberine treatment led us to speculate that programmed cell death could be oncosis, characterized by a rapid change in the cell volume, a decrease in ATP level, an increase in Na^+^ and Ca^2+^ ions, and increased water content [37]. Thus, it is possible that rapid loss of ATP reduces the ability of colon cancer cell lines to maintain ATP levels at sufficient levels to drive caspase-3 activation and apoptosis. Consequently, when ATP levels decrease, cells lose the ability to appropriately modulate plasma membrane ion balance and undergo oncosis-mediated cell death [38]. To examine this hypothesis, the RealTime-Glo Annexin V Apoptosis and Necrosis Assay was performed to analyze the response of the SW620 cell line treated with 10 and 50 µM of liposomes and its free form over 48 h. In this experiment, compounds that induce apoptosis (such as doxorubicin used in our experiment as a positive control) produce time- and dose-dependent increases in luminescence—in early apoptosis, phosphatidylserine (PS) dislocates from the inner side of the cell membrane to the exterior and can then bind to the annexin V fusion protein, which precedes a temporal increase in fluorescence due to secondary necrosis (loss of membrane integrity). Figure 13a shows that berberine reduced the population of live colon cancer cells by a decrease in luminescence compared to untreated cells. In the meantime, measurement of fluorescence showed that a high concentration of free berberine increased the number of cells, with disturbances in the cell membrane integrity (Figure 13b). Based on this observation, it could be said that treatment with berberine led to an increase in the number of late-apoptotic/necrotic cells. The berberine-loaded liposomes with vitamin C worked in an almost identical manner.

### 2.9. Oncosis Induction by Liposomal Berberine Correlated with Induction of Mitochondrial Respiration Dysfunction and Decreased ATP Production Efficiency

**Figure 14:**
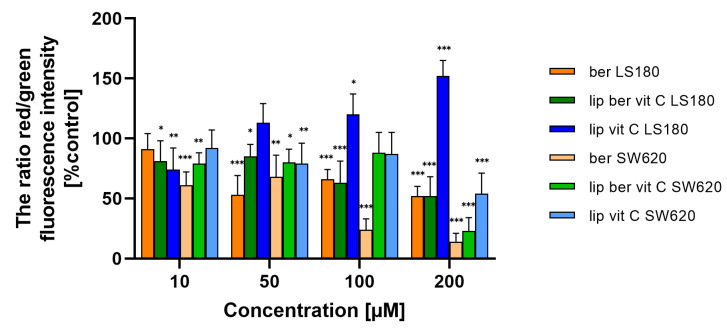
Δψm changes in colon cancer cells after treatment of berberine loaded liposomes, blank-liposomes, and free berberine (10, 50, 100, or 200 µM) for 24 h. Δψm was determined by fluorometry. The ratio of red to green fluorescence intensity of untreated cells (control) was considered to be 100% of the Δψm level. Data represent the mean ± SD of three independent biological replicates. Statistical significance was determined using a one-way ANOVA (Dunnett’s modification) test. *** *p* < 0.001; ** *p* < 0.002; * *p* < 0.033.

We next assessed whether liposomal berberine-induced cell death in colon cancer cells shared the principal biochemical features previously described for oncosis-mediated cell death; these include changes in the function of the mitochondria, which implicates a decrease in Δψm, sustained Ca^2+^ influx, ROS generation, and GSH decrease. First, changes in Δψm due to liposomal berberine treatment were examined by JC-1 staining [39]. Figure 14 shows that both colon cancer cell lines were characterized by a dose-dependent depolarization of Δψm after 24 h of treatment, either with berberine or its liposomal formulation. It was previously reported that caspase-3-negative cell lines were characterized by a similar fold change in the decrease of Δψm after 48 h of treatment [38]. Figure S15 shows that both colon cancer cell lines were characterized by a dose-dependent hyperpolarization of Δψm after 48 h of treatment, either with berberine or its liposomal formulation.

**Figure 15:**
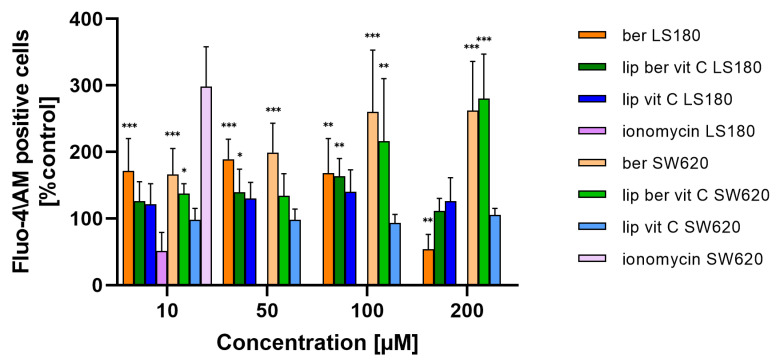
Changes in Ca^2+^ level in colon cancer cells after treatment with free berberine, blank liposomes, or berberine-loaded liposomes (10, 50, 100, or 200 µM) for 24 h. RFU of untreated cells (control) was considered to be 100% of the Ca^2+^ level. Data represent the mean ± SD of four independent biological replicates. Statistical significance was determined using a one-way ANOVA (Dunnett’s modification) test. *** *p* < 0.001; ** *p* < 0.002; * *p* < 0.033.

Consistent with the assumption of a correlation between loss of plasma membrane integrity and increased intracellular Ca^2+^, we also assessed whether liposomal berberine-induced cell death in colon cancer cells includes a sustained calcium influx following ATP depletion. Changes in intracellular Ca^2+^ levels due to liposomal berberine treatment were examined by Fluo-4/AM staining [40]. Both colon cancer cell lines were characterized by an increase in Ca^2+^ levels in the cytoplasm after 24 h of treatment with berberine or its liposomal formulation (Figure 15 and Figure S16), indicating the relevance of calcium in the cell death induced by berberine. As a positive control, ionomycin was used. Interestingly, LS180 cells were characterized by a decrease in the amount of Ca^2+^ ions present in the cytoplasm after ionomycin treatment. Based on a previously reported study [41], the most likely explanation for the observed effect could be related to the instability of ionomycin in the LS180.

**Figure 16:**
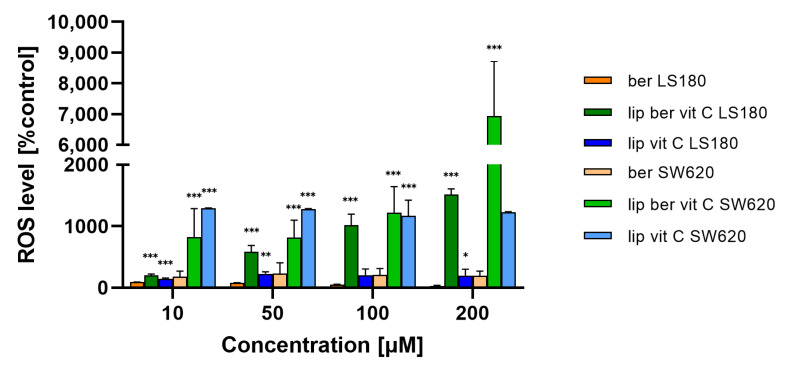
Increased ROS level in colon cancer cells after treatment with free berberine, blank liposomes, or berberine-loaded liposomes (10, 50, 100, or 200 µM) for 24 h. Luminescence of untreated cells (control) was considered to be 100% of the ROS level. Data represent the mean ± SD of two independent biological replicates. Statistical significance was determined using a one-way ANOVA (Dunnett’s modification) test. *** *p* < 0.001; ** *p* < 0.002; * *p* < 0.033.

Mitochondria are the major organelles for ROS elimination, and mitochondrial impairment decreases the ability of cells to eliminate ROS. In an antioxidation system, (glutathione) GSH plays a central role in eliminating ROS. As shown in Figure 16 and Figure S17, the treatment of colon cancer cells with a berberine liposomal formulation with vitamin C markedly increased H_2_O_2_-induced cell death compared to the untreated cells.

**Figure 17:**
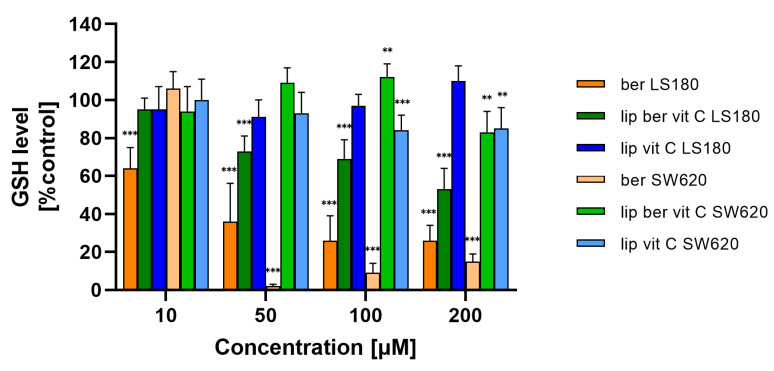
Decreased GSH level in colon cancer cells after treatment with free berberine, blank liposomes, or berberine-loaded liposomes (10, 50, 100, or 200 µM) for 24 h. Luminescence of untreated cells (control) is considered to be 100% of the GSH level. Data represent the mean ± SD of three independent replicates. Statistical significance was determined using a one-way ANOVA (Dunnett’s modification) test. *** *p* < 0.001; ** *p* < 0.002.

Moreover, the obtained results showed a correlation between the increase in ROS levels and a decrease in GSH levels in colon cancer cells treated with the liposomal formulation of berberine. These results indicate that berberine induces oxidative stress (Figure 17 and Figure S18).

### 2.10. Liposomal Berberine Treatment Induces CRT Exposure in a Colon Cancer Cell Line

**Figure 18:**
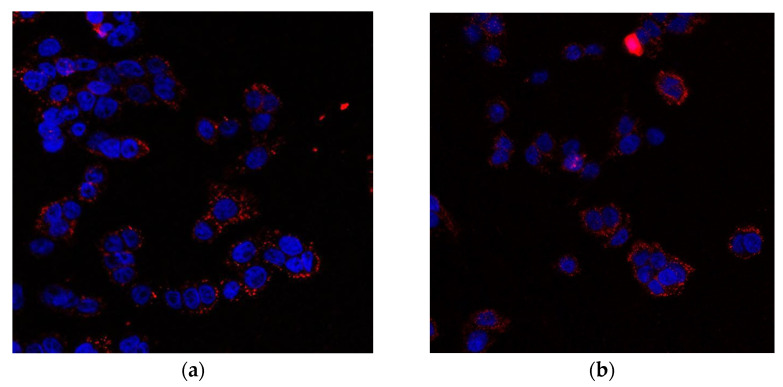
Microscopic photo of SW620 cells (**a**) after treatment with 10 µM berberine (**b**) after treatment with 10 µM berberine-loaded liposomes with vitamin C. The cell nuclei were stained with DAPI and cell surface CRT bound by anti-CRT antibody recognized by Alexa Fluor 647 conjugated secondary antibody.

As CRT is one of the principal molecules necessary to determine the ICD [42], we assessed its surface exposure in colon cancer cells. Figure 18 shows that treatment of SW620 cells with liposomal berberine led to an increase in CRT on their cell surface, as analyzed by confocal microscopy. Figure S19 presents increased CRT cell surface exposure after berberine stimulation on the monocyte-derived macrophage THP-1 cell surface.

### 2.11. Release of the High Mobility Group Box 1 Protein (HMGB1) and ATP to the Extracellular Environment

**Figure 19:**
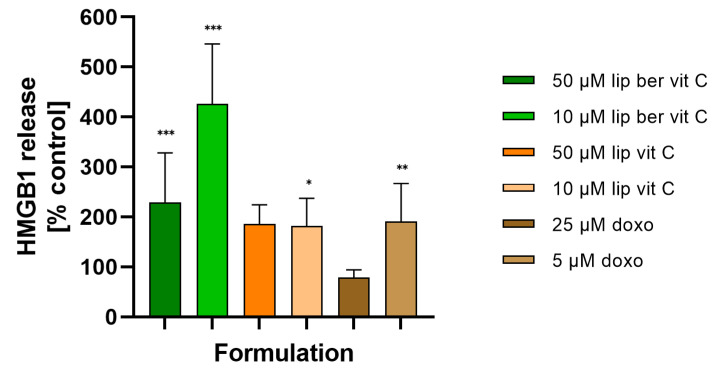
HMGB1 release from SW620 cells after treatment with free berberine, blank liposomes, or berberine-loaded liposomes (10 and 50 µM) for 24 h. Doxorubicin was used as a positive control. Luminescence of untreated cells (control) was considered to be 100% of the HMGB1 level. Data represent the mean ± SD of five independent biological replicates. Statistical significance was determined using a one-way ANOVA (Dunnett’s modification) test. *** *p* < 0.001; ** *p* < 0.002; * *p* < 0.033.

In response to immunogenic chemotherapeutics, dying cells release HMGB1 protein and secrete ATP into the extracellular environment, stimulating an immunogenic response. To verify whether HMGB1 release and ATP secretion play a role in the mechanism of action of berberine, we analyzed HMGB1 release and extracellular ATP secretion in the cell culture supernatants of SW620 cells treated with berberine and, for comparative purposes, also doxorubicin. HMGB1 release was detected by the Lumit HMGB1 Human Immunoassay. Using 10 µM liposomal berberine, HMGB1 release was four-fold higher compared to the untreated cells, whereas using other concentrations of compounds, release was two-fold higher compared to the untreated cells (Figure 19).

**Figure 20:**
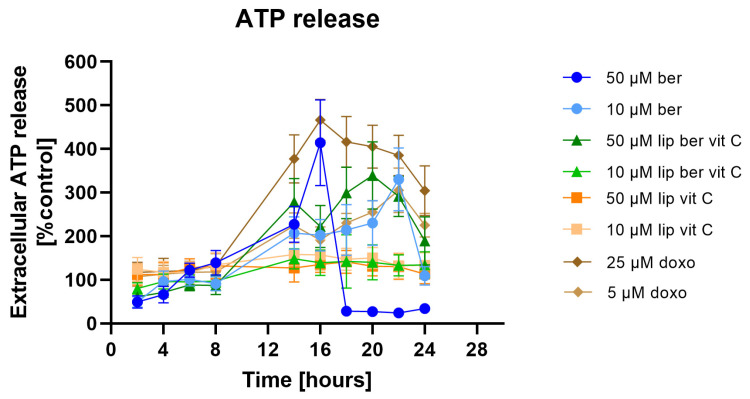
Extracellular ATP release from SW620 cells after treatment with free berberine, blank liposomes, or berberine-loaded liposomes (10 and 50 µM) for 24 h. Doxorubicin was used as a positive control. Luminescence of untreated cells (control) was considered to be 100% of the ATP level. Data represent the mean ± SD of four independent replicates.

The extracellular ATP from cell supernatants was analyzed with the RealTime-Glo Extracellular ATP Assay, finding that in the supernatants of berberine-treated cells at 10 and 50 µM, the presence of ATP significantly increased (Figure 20). Similar results were obtained for 50 µM berberine-loaded liposomes with vitamin C.

### 2.12. In Vitro Phagocytosis Assay

**Figure 21:**
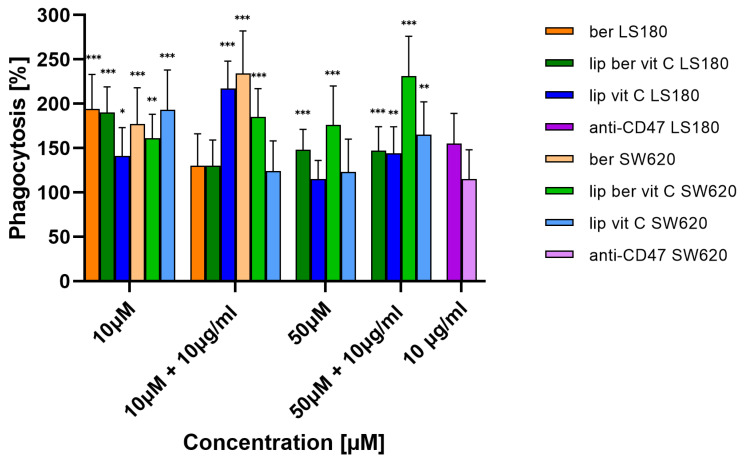
Phagocytosis of colon cancer cells after treatment with free berberine, blank liposomes, or berberine-loaded liposomes (10 and 50 µM) for 24 h. After this time, the 10 µg/mL anti-CD47 antibody was added for a further 2 h co-incubation with monocyte-derived macrophages THP-1. Data represent the mean ± SD of four independent biological replicates. Statistical significance was determined using a one-way ANOVA (Dunnett’s modification) test. *** *p* < 0.001; ** *p* < 0.002; * *p* < 0.033.

For determining the phagocytic capacity of monocyte-derived macrophages in berberine-treated cancer cells, a fluorescent plate-based assay was performed. Colon cancer cells were pre-treated for 24 h with berberine-loaded liposomes, blank liposomes, and a free form of berberine, followed by co-incubation with human THP-1 macrophages. Figure 21 shows the increased uptake of fluorescently labeled treated colon cancer cells compared to untreated cancer cells. Moreover, a combination of berberine and anti-CD47 antibodies showed stronger engulfment of SW620 cells by macrophages than a single agent.

**Figure 22:**
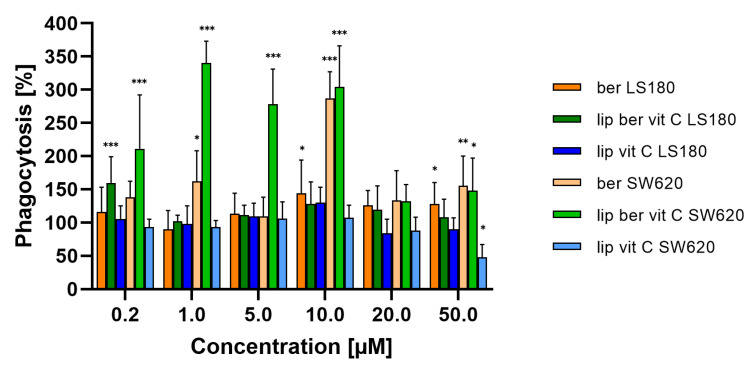
Phagocytosis of colon cancer cells after macrophage pre-treatment with free berberine, blank liposomes, or berberine-loaded liposomes (10 and 50 µM) for 24 h. After this time, colon cancer cells were added for a further 2 h co-incubation with monocyte-derived macrophages THP-1. Data represent the mean ± SD of three independent biological replicates. Statistical significance was determined using a one-way ANOVA (Dunnett’s modification) test. *** *p* < 0.001; ** *p* < 0.002; * *p* < 0.033.

To assess the phagocytic efficacy of berberine-treated macrophages, monocyte-derived macrophages THP-1 were pre-treated for 24 h with berberine-loaded liposomes, blank liposomes, and a free form of berberine, followed by co-incubation with colon cancer cells. THP-1 macrophages treated with berberine showed 3-fold greater phagocytic efficiency of SW620 cells versus non-treated macrophages (Figure 22).

**Figure 23:**
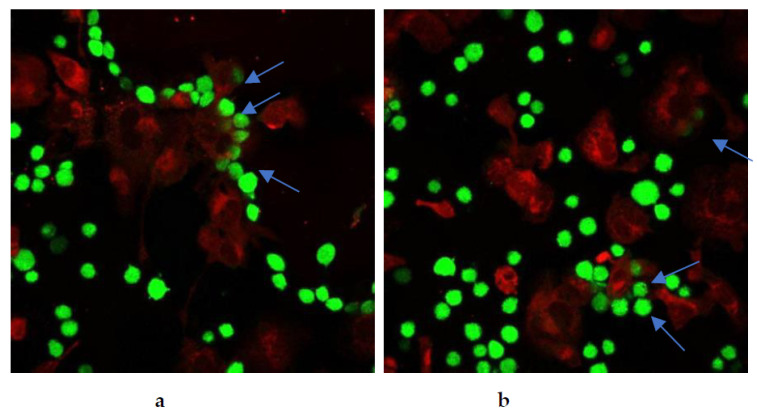
Berberine restored phagocytosis of SW620 cells after cell pre-treatment with either (**a**) 10 µM berberine or (**b**) 50 µM berberine-loaded liposomes with vitamin C for 24 h. The arrows indicate engulfment of colon cancer cells by macrophages.

**Figure 24:**
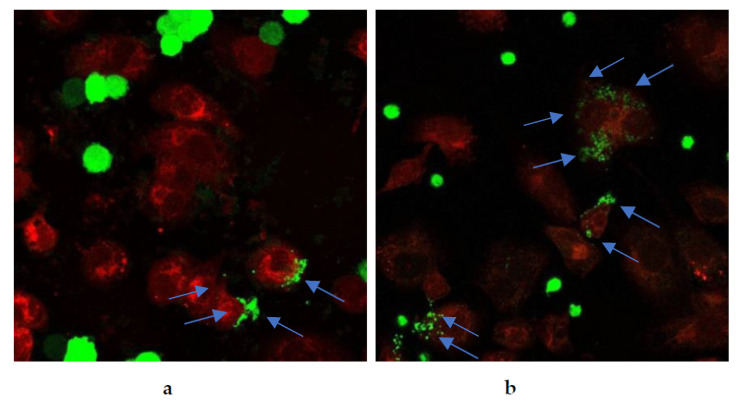
Berberine restored phagocytosis of SW620 cells after macrophage pre-treatment with either (**a**) 10 µM berberine or (**b**) 50 µM berberine-loaded liposomes with vitamin C for 24 h. The arrows indicate engulfment of colon cancer cells by macrophages.

Restoration of cancer cell phagocytosis following berberine pretreatment was also verified by confocal microscopy (Figure 23 and Figure 24). Successful phagocytosis occurred in those CellTrace Far-Red-labeled macrophages in which CellTrace CFSE-labeled cancer cells were ingested or partially ingested.

## 3. Discussion

In our work, we used HSPC, Chol, and DSPE-PEG_2000_ in a molar ratio of 5.5:4.0:0.5. The choice of such a lipid composition was dictated by the properties of the individual lipids. The addition of cholesterol fluidizes the lipid bilayer composed of HSPC, which is characterized by a high transition temperature and a stronger order parameter [43,44,45]. In addition, DSPE-PEG_2000_ was added to modify the surface of liposomes in order to have a formulation exhibiting a prolonged circulation time so as to improve liposome accumulation in cells and potentiate the EPR effect [43]. Hydration of thin lipid films was performed by using various aqueous solutions to generate pH gradients, namely vitamin C, citric acid, and ammonium sulfate. The physicochemical characterization of liposomes, such as size and shape, are vital parameters to deliver improved biodistribution and prolonged pharmacokinetics of encapsulated drugs [46,47]. Table 1 summarizes the physicochemical parameters of all of the tested liposomal formulations. The preparations had a small homogeneous size, a low PDI index, indicating good homogenization of liposomes during extrusion, and no aggregation or fusion processes occurring after liposome preparation. Our formulations showed the ability to encapsulate with more than 90% of berberine when a drug-to-lipid weight ratio up to 0.1 was used. Other studies have shown high encapsulation efficiency of berberine inside liposomes when a drug-to-lipid ratio of 1:20 was used [48,49]. During the conducted experiments for optimal conditions selection for berberine encapsulation, we observed prolonged periods of incubation of berberine with liposomes at 60 °C, followed by its rapid leakage. Most likely, the observed berberine efflux is related to the better solubility of the ascorbate-berberine complex and the possible partial destabilization of the pH gradient at high temperatures, which facilitated its free diffusion through the membrane. A similar temperature dependency was described previously [48]. Based on the cryogenic transmission electron micrographs, berberine does not form a poorly soluble precipitate in the form of elongated bundles (“coffee-bean” structure) inside liposomes, which was previously observed for other drugs encapsulated in such a lipid formulation [35,50]. According to the logP value (−1.28) [29], berberine is a relatively hydrophilic compound, leading to its presence inside liposomes, partially in a crystallized state and partially in the form of a soluble salt with a compound generating a pH gradient. A similar lack of precipitation inside liposomes was previously described by our working team [31]. Our developed formulations are stable in suspension when stored at 4 °C for 1 year. Neither liposome aggregation nor an increase in the PDI were observed during liposome storage. Supplementary Table S1 summarizes the physicochemical parameters of all of the tested liposomal formulations. Phospholipids with a high phase transition temperature, such as HSPC, are characterized by high membrane stability and lower drug-release rates [51,52]. Perhaps the slight leakage of berberine from liposomes was connected with the higher solubility of both salts (berberine-ascorbic acid and berberine-ammonium sulfate salt) compared to berberine-citric acid salt, which was supported by the few noticeable elongated bundles inside the liposomes containing citric acid in the cryo-TEM micrographs. Liposomal berberine formulations showed time-dependent gradual retention of berberine in the plasma, which coincides with the previous observation during the long-term stability experiment. One explanation could be the high affinity of HSPC for plasma proteins, which increases the probability of liposomes’ destabilization and berberine leakage. According to the literature, the order of decreasing affinity of phospholipids to plasma proteins is as follows: HSPC:DPPC:DMPC [53].

Among the designed formulations, the vitamin C-driven liposomal berberine formulation was found to have high long-term stability in vitro, low drug release in plasma in vitro, and the highest cytotoxic activity for colon cancer cell lines. Vitamin C is able to synergize with all drugs whose mechanism of action is related to free radical formation. According to the literature, ascorbate has the ability to release iron ions from the plasma storage protein ferritin, making iron ions catalytically available for free-radical reactions [54]. Using ascorbate as a pH-generating gradient agent, we could selectively increase the oxidative stress of the cancer cells, which was proved by the ROS-Glo™ H_2_O_2_ Assay performed. The mechanism of action of our developed drug loaded into vesicles model involved the induction of the H_2_O_2_-dependent cell death pathway. The observed effect may be related to the occurrence of potential synergistic action between both substances, leading to the generation of H_2_O_2_. One of berberine’s molecular targets is NADH dehydrogenase, which is responsible for converting NADH molecules to NAD^+^. NAD^+^ is required as both a substrate and a cofactor for a large number of metabolic enzymes, and its depletion can impact flux through pathways that are dependent on these enzymes. As such, a range of metabolic perturbances likely contribute to the observed loss of cellular ATP following NAD^+^ depletion [38]. Moreover, another study reported that a high dose of vitamin C via H_2_O_2_ depletes the level of NAD^+^, thus preventing the flow of energy from the glycolysis process and the TCA cycle and consequently reducing the production of ATP [34,55]. Perhaps the observed lack of cytotoxic activity of the liposomal vitamin C towards colon cancer cell lines is most likely due to the low concentration used. The hydrogen-peroxide-dependent cytotoxicity toward colon cancer cells in vitro of high-dose intravenous ascorbic acid is dependent on the production of H_2_O_2_, which takes place when ascorbate is at millimolar levels [54]. The above hypothesis is confirmed by our GSH level results, which show that the liposomal form of vitamin C does not affect the GSH level. Moreover, similar results were presented by Legut, M., et al., where tested melanoma cancer cell lines were sensitive to mitoxantrone in a dose-dependent manner, while blank liposomes with vitamin C showed negligible toxicity, which was approximately the same level for both cell lines [32]. 

ATP is critical for the execution of apoptosis, and once ATP levels are depleted, the cells can switch from undergoing apoptosis to oncosis. Consequently, when ATP levels decrease, cells lose the ability to appropriately modulate plasma membrane ion balance and undergo oncosis-mediated cell death [38]. This study showed that berberine-induced colon cancer cell death was characterized by a lack of caspase activity, decreased Δψm, and markedly reduced ATP levels.

Cellular ROS levels affect redox status and metabolism [55]. Eukaryotic cells have developed a system-based protective mechanism for buffering the level of cysteine residues and forming disulfide bridges for neutralizing ROS [56]. H_2_O_2_ can change the ratio of oxidized glutathione (GSSG) and GSH to a more oxidized state since H_2_O_2_ is reduced to water (H_2_O) by glutathione peroxidase (GPx) [55]. Treatment of colon cancer cells with a berberine liposomal formulation with vitamin C markedly decreased GSH levels compared to the untreated control. A likely explanation could be that one of vitamin C’s molecular targets is glyceraldehyde 3-phosphate dehydrogenase (GAPDH). An increased amount of GLUT1 receptors in colon cancer cells leads to efficient transport of the oxidized form of ascorbic acid (dehydroascorbic acid) and its accumulation in cells, where it is reduced to ascorbic acid. The above process lowers GSH levels, leading to the accumulation of ROS, which interact with the cysteine in the active center of GAPDH to deactivate it [57].

We believe that the observed alterations are connected with ICD. Intracellular oxidative stress is a key factor in the activation of ICD. Until now, ICD was thought to arise from ER stress caused by ROS accumulation. The molecular bridge regulating contact between the ER and mitochondria is called mitochondria-associated membranes (MAMs) [5]. The inositol trisphosphate receptor (IP_3_R) present in the ER, together with the ryanodine receptor (RyR), is responsible for the formation of microdomains containing a high concentration of Ca^2+^ (relative to the rest of the cytosol) needed for active transport of Ca^2+^ ions into the mitochondrial matrix via the mitochondrial calcium uniporter (MCU) [58,59]. Since the ER plays a key role in modulating Ca^2+^ mobilization, we considered the potential role of Ca^2+^ in berberine-mediated ICD. We observed that berberine treatment induced a significant increase in Ca^2+^ levels in the cytosol compared to the untreated control. Optimal-functioning proteins found in the ER require an environment containing large amounts of Ca^2+^ ions. One of the effects of the increased amount of ROS in the mitochondria may be the oxidation of a key thiol residue in the RyR receptor, causing the release of Ca^2+^ ions from the ER and consequent ER stress [60]. Thus, leakage of the above ions affects the CRT conformation and is responsible for its translocation to the surface of the dying cells [58,59,61]. Surface exposure of CRT, a protein that enables phagocytes to efficiently engulf dead cells [42,62], observed in berberine-treated colon cancer cells appears, in our view, to be the consequence of continuous liberation of Ca^2+^ from the ER. Based on our results, we inferred that liposomal berberine could be an ICD inducer; thus, we assessed the release of other DAMPs (including extracellular ATP and HMGB1 protein) in colon cancer cells.

In an in vitro phagocytosis assay, we demonstrated that berberine pre-treatment for 24 h increased the phagocytosis of SW620 cells by macrophages. Moreover, co-treatment of berberine and anti-CD47 antibody showed more potent engulfment of SW620 cells by macrophages than as a single agent used. Recently, other studies reported CD47 downregulation in DLBCL cells following berberine treatment via modulating ERK1/2 phosphorylation and c-Myc expression [63]. Through targeting CD47 overexpressed on DLBCL cells, berberine restored the phagocytosis of macrophages [64]. Moreover, the authors showed that treatment of tumor cells with berberine increased the efficiency of phagocytosis in the presence of an anti-CD47 antibody. Additionally, it has been reported that the mitochondrial p-ERK level in T98G glioma cells was down-regulated after berberine treatment for 48 h, which was in line with the significant reduction of ATP levels [65]. Based on the data obtained from the available literature, we hypothesize that SW620 cells characterized by a high level of CD47 expression were down-regulated by berberine treatment, leading to increased phagocytosis [64,66].

Berberine plays a vital role in immunogenic colon cancer cell death, but its role in macrophage stimulation remains unclear. In an in vitro phagocytosis assay, we demonstrated that macrophage pre-treatment with up to 10 µM berberine concentration for 24 h increased phagocytosis in SW620 cells. The obtained results are in line with published data from over 40 years ago, which have shown that a very low concentration of berberine (0.05–5 μg/mL) leads to increased cytostasis of cancer cells [23]. Galectins are galactoside-binding proteins overexpressed in colorectal cancer and are involved in regulating its development, progression, and metastasis [67]. They recognize glycans containing N-acetylglucosamine, composed of galactose and N-acetylglucosamine [68]. Several years ago, it was shown that CRT occurring on the surface of macrophages is involved in the removal of live cancer cells via binding to asialoglycoproteins present on the surface of tumor cells composed of N-acetylglucosamine molecules, mannose, and galactose [29,39,42]. We believe that the observed enhanced phagocytosis of SW620 cells is associated with overexpression of galectins and increased amounts of asialoglycoproteins, which are ligands for calreticulin present on the macrophage surface.

## 4. Materials and Methods

### 4.1. Materials

Hydrogenated Soy Phosphatidylcholine (Phospholipon 90H, HSPC, purity ≥90.0%) and N-(carbonyl-methoxypolyethylene glycol-_2000_)-1,2-distearoyl-sn-glycero-3-phosphoethanolamine (LIPOID PE 18:0/18:0—PEG_2000_, DSPE-PEG_2000_) were purchased from Lipoid GmbH (Ludwigshafen, Germany). Cholesterol (Chol) was purchased from Transferra Nanosciences Inc. formerly known as Northern Lipids Inc. (Burnaby, close to Vancouver, BC, Canada). Chloroform, isopropanol, cyclohexane, methanol, sodium dihydrogen phosphate, disodium hydrogen phosphate, sodium bicarbonate, sodium carbonate, sodium chloride, dimethyl sulfoxide (DMSO), ascorbic acid, citric acid, ammonium sulfate, acetic acid, trichloroacetic acid (TCA), and Tris-base were purchased from Avantor Performance Materials Poland S.A. (Gliwice, Poland). Berberine chloride (hereinafter referred to as berberine, purity ≥ 98.0%), Sephadex G-50 Fine, Sepharose CM-4B, sulforhodamine B, phorbol-12-myristate-13-acetate (PMA), paraformaldehyde (PFA), and DAKO mounting medium were obtained from Merck Life Science Sp.z.o.o., an affiliate of Merck KGaA, Darmstadt, Germany (Poznan, Poland). Cell culture medium (EMEM, PBS buffer, and Trypsin-EDTA) was purchased from Lonza (Basel, Switzerland). Cell culture media RPMI 1640, L-15, MEMα, Fetal Bovine Serum (FBS), and NaHCO_3_ were purchased from Biowest (Nuaillé, France). Nuclepore Whatman filters 0.2 and 0.1 µm were obtained from Cytiva (distributor-Lab-System-Service, Szczecin, Poland). CellTrace Far-Red, CellTrace Green, Fluo-4 AM (cat.no. F14201), Gibco™ Antibiotic-Antimycotic, and 2 mM glutamine were purchased from Life Technologies Polska Sp. z o. o. (Warsaw, Poland). Anti-Calreticulin antibody—ER Marker (ab2907), goat Anti-Rabbit IgG H&L (Alexa Fluor^®^ 647) (ab150079), and Fluoroshield Mounting Medium with DAPI were obtained from Abcam (distributor—Symbios Sp. z o. o, Gdansk, Poland). The anti-CD47 antibody (cat.no. 556044) was purchased from Becton Dickinson Polska Sp. z o.o. (Warsaw, Poland). JC-1 (cat.no. 15003) was purchased from Cayman Chemical (Michigan, MI, USA). CellTiter-Glo Luminescent Cell Viability Assay, Caspase-Glo 3/7 Assay, Caspase-Glo 9 Assay, Caspase-Glo 8 Assay, ROS-Glo H_2_O_2_ Assay, RealTime-Glo Annexin V Apoptosis and Necrosis Assay, RealTime-Glo Extracellular ATP Assay, Lumit HMGB1 Human Immunoassay, and GSH-Glo Glutathione Assay were purchased from Promega GmbH (Walldorf, Germany). Acetonitryle, orthophosphoric acid, and HPLC water with analytical grade were purchased from Merck Life Science Sp.z.o.o., an affiliate of Merck KGaA, Darmstadt, Germany (Poznan, Poland). DAKO mounting medium was purchased from Agilent Technologies, Inc. (Supplier-Altium International z.o.o, Warsaw, Poland).

### 4.2. Preparation of Blank Liposomes

10 mg/mL chloroform stock solutions of lipids (HSPC/Chol/DSPE-PEG_2000_) were prepared, and 40–60 mg of lipids were then transferred to borosilicate glass tubes to obtain molar ratios of lipids (5.5:4.0:0.5). The solvent was then evaporated using a nitrogen stream. Obtained thin lipid films were dissolved in 1 mL of cyclohexane with 0.1% (*v*/*v*) methanol, frozen in liquid nitrogen, and freeze-dried overnight at low pressure using a Savant Modulyo D Bench Freeze Dryer-Lyophilizer (Thermo Electron Corporation, formerly known as Savant, Waltham, MA, USA). After lipid hydration with 1.5 mL of 300 mM ascorbic acid, 300 mM citric acid, or 300 mM ammonium sulfate in a water bath at 64 °C (POLSONIC Palczynski Sp. J. Warsaw, Poland), a freeze-and-thaw (FAT) procedure (repeated 10 times) for the obtained multilamellar vesicles was performed, followed by extrusion 10 times repeated through Nuclepore polycarbonate filters (pore sizes of 200 and 100 nm) on a 10 mL Thermobarrel Lipex Extruder at 64 °C (Transferra Nanosciences Inc. Burnaby, close to Vancouver, BC, Canada). Non-encapsulated salt was removed from salt-containing liposomes by size exclusion chromatography on a Sephadex G-50 fine (1 × 20 cm) column equilibrated with 0.9% sodium chloride (NaCl) solution. Phospholipid concentration was determined by ammonium ferrothiocyanate assay [69] on the Shimadzu UV 2401 PC spectrophotometer (Shimadzu Corp., Kyoto, Japan) at 485 nm. Liposome size and PDI were determined by dynamic light scattering with the Zetasizer Nano ZS (Malvern Panalytical Ltd., Malvern, UK). Liposomes were diluted by adding 50 µL samples to 1 mL of 0.9% NaCl prior to measurements.

### 4.3. Optimization of the Berberine Active Loading inside the Liposomes

A pH gradient was generated by adding external buffer in the proportion 1:9 (*v*/*v*) to obtain a final concentration of 20 mM in a mixture of liposomes containing a salt gradient with an aqueous solution of berberine (4 mg/mL in Mili-Q water). Due to poor water solubility, berberine solution was dissolved in a Julabo TW 12 water bath at 60–64 °C (Julabo GmbH, Seelbach, Germany). Phosphate buffers of pH 7.5 and 8.5, as well as carbonate buffer, pH 9.2, were used in the experiments to determine the influence of the external pH on berberine EE%. Drug-to-lipid ratios (*w*/*w*) of 1:5, 1:10, and 1:20 were used to determine the EE% at different ratios. To determine the kinetics of drug encapsulation, liposomal samples were taken after loading for 5, 10, 15, 30, or 60 min at 50 °C or 60 °C.

### 4.4. Optimization of the Berberine Active Loading inside the Liposomes

The non-encapsulated berberine was removed from the berberine-containing liposomes using size-exclusion chromatography on a Sephadex G-50 mini-column (5.5 × 70 mm) equilibrated with 0.9% NaCl. 100 μL liposome samples were placed on the column, and free berberine was separated from berberine-containing liposomes. Then, the liposome fraction was collected, and the concentration of lipid and berberine was determined to calculate the encapsulation efficiency. The phospholipid concentration was determined by an ammonium ferrothiocyanate assay on the Shimadzu UV 2401 PC spectrophotometer at 485 nm. The berberine concentration was measured with high-performance liquid chromatography on the Waters 600 HPLC System (Waters Corporation, Milford, MA, USA) after liposome dissolution in isopropanol. The chromatographic conditions were as follows: Waters XBridge RP18 column (250 × 4.6 mm, 5 μm), acetonitrile/0.05% orthophosphoric acid in water (50:50, *v*/*v*) mobile phase with a flow rate of 1 mL/min. The detection was done using a Waters 996 UV-vis detector at λ = 345 nm. The acquired data were processed using Empower 2 (build 2154) software (Waters Corporation, Milford, MA, USA). The relative amount of berberine was calculated from the appropriate calibration curve. The EE % was calculated according to the following equation:

EE = (a/b) × 100%,(1)

where the value is expressed by the equation:

a = berberine/lipid [mg/mL](2)

And the b value is expressed by the equation:

b = initial (berberine/lipid) [mg/mL](3)

a is the measured amount of berberine in the liposome suspensions after passing through the Sephadex G-50 fine column, and b is the measured amount of berberine in the liposome suspension before passing through the Sephadex G-50 fine column.

### 4.5. Cryo-Transmission Electron Microscopy

For cryo-TEM experiments, berberine-loaded liposomes composed of HSPC/Chol/DSPE-PEG_2000_ (5.5:4.0:0.5 mol/mol) were prepared with a drug-to-lipid ratio (*w*/*w*) of 1:10. Liposome formulations were prepared to a total lipid concentration of 10 mg/mL. Copper grids (S7/2 Cu 400 mesh, porous carbon membrane, Quantifoil Micro Tools GmbH, Jena, Germany) were prepared according to standard procedures. After placing a drop of sample on the grid, use filter paper to remove most of the liquid, leaving the film stretched over the wells. Samples were immediately snap-frozen in liquid ethane, stored in liquid nitrogen at 90 K, and loaded into cryogenic sample holders (D262; Gatan Inc., Pleasanton, CA, USA). Samples were transferred to a Leo 912 Omega microscope (Carl Zeiss, Oberkochen, Germany) and examined at 100 K. Digital images were recorded using a slow-scan charge-coupled device (SSCCD) photography system (Proscan HSV 2, Oxford Instruments, Abingdon, WA, USA) under low-dose conditions, i.e., using a minimum-dose focusing device.

### 4.6. Long-Term Berberine Retention in Liposomes

Berberine was encapsulated in HSPC/Chol/DSPE-PEG_2000_ (5.5:4.0:0.5 mol/mol) liposomes at the 1:10 substance-to-lipid ratio (*w*/*w*) using the following gradients: 300 mM ammonium sulfate, 300 mM ascorbic acid, and 300 mM citric acid. The active loading process was carried out at 50 °C for 15 min. non-encapsulated berberine was removed on a Sephadex G-50 fine column (1 × 20 mm) equilibrated with a 0.9% NaCl solution. Then the liposomal samples were diluted to obtain a 4 mM liposomal concentration with a PBS solution containing 0.01% NaN_3_ and stored at 4 °C. At selected time intervals, liposomal samples were placed on Sephadex G-50 mini columns (5.5 × 70 mm) equilibrated with PBS to separate the liposomal fraction from the released berberine. Then the berberine and lipid concentrations were measured in the collected liposomal fractions, and the resulting substance-to-lipid ratio was compared with the initial values as described above for the determination of EE (%).

### 4.7. Stability of Berberine-Loaded Liposomes in the Presence of Human Plasma In Vitro

Berberine was encapsulated in HSPC/Chol/DSPE-PEG_2000_ (5.5:4.0:0.5 mol/mol) liposomes at a 1:10 substance-to-lipid ratio (*w*/*w*) using the following gradients: 300 mM ammonium sulfate, 300 mM ascorbic acid, and 300 mM citric acid. The active loading process was carried out at 50 °C for 15 min. The liposomal samples were diluted to obtain a total lipid concentration of 4 mM. Each liposomal suspension was then mixed with fresh human plasma in a 1:1 (*v*/*v*) ratio to a final lipid concentration of 2 mM. The liposomes were incubated in a Julabo TW 12 water bath at 37 °C for 24 h. At 15 min, 30 min, 1 h, 1.5 h, 2 h, 4 h, 6 h, 12 h, and 24 h, 200 μL samples were placed on Sepharose CM-4B mini columns pre-equilibrated with 0.9% NaCl solution to separate the liposomal fraction from the released berberine (free and protein bound). Then the berberine and lipid concentrations were measured in the collected liposomal fractions, and the resulting substance-to-lipid ratio was compared with the initial values as described above for the determination of EE (%).

### 4.8. Cell Lines

All cell lines were obtained from our in-house culture cell bank (the original source was ATCC). LS180 and SW620 colon cancer cell lines were cultured in EMEM and L-15, respectively. The normal colon CCD112CoN cell line was cultured in MEMα, and the human leukemia monocytic cell line THP-1 was cultured in RPMI 1640. All growth media were supplemented with 10% heat-inactivated FBS, 2 mM glutamine, and 1% antibiotics. Additionally, L-15 growth medium was supplemented with 2.75% NaHCO_3_. The cells were cultured at 37 °C in an incubator with 5% CO_2_.

### 4.9. Cell Proliferation Measurements

A Sulforhodamine B (SRB) assay was conducted to measure the effects of berberine on the proliferation of colon cells, as described in detail previously [70]. In brief, LS180, SW620, and CCD112CoN cells were seeded into transparent 96-well plates in 100 µL of the appropriate growth medium per well and incubated overnight. The number of LS180 cells was 2 × 10^3^/well, while the other two lines were seeded at 4 × 10^3^ cells/well. The next day, cells were treated with different concentrations of berberine, blank liposomes, and berberine-loaded liposomes (5, 10, 25, 50, 100, and 200 μM), followed by further incubation for 24, 48, and 72 h. After treatment, cells were fixed by adding 50 µL/well of 50% TCA for 1 h at 4 °C. Plates were washed five times with water to remove TCA and dried. This was followed by 50 μL of 0.4% SRB solution in 1% acetic acid (*w*/*v*) staining for 30 min at room temperature (RT) in the dark. Unbound SRB was removed by washing each well five times with 1% acetic acid and drying. The bound dye was dissolved in 150 μL/well of 10 mM unbuffered Tris base, and then the plates were mixed using a MixMate plate shaker (Eppendorf, Poland) at 1000 rpm for 2.5 min. The absorbance was measured using a spectrophotometer (Asys UVM 340 microplate reader, Biogenet, Jozefow, Poland) at 540 nm. The mean absorbance of non-treated cells served as the reference value for calculating the percentage of cellular viability. The assay was carried out in triplicate. Culture medium without cells was used as a background control (blank) and was subtracted from the other measurements. Cell viability was calculated as follows:

Cell viability = (A_540_ treated cells − A_540_ medium/A_540_ untreated cells − A_540_ medium) × 100% (4)

### 4.10. Determination of Oxidative Stress

ROS generation as well as GSH levels were monitored by using the ROS-Glo H_2_O_2_ assay and the GSH-Glo assay following the manufacturer’s instructions, respectively. In brief, LS180 and SW620 cells were plated into white 96-well culture plates (Scholagene) at a density of 1.5 × 10^4^ cells/well and allowed to adhere overnight. Cells were then treated with liposomal berberine and free berberine in a dose-dependent manner and incubated for the selected time points—24 and 48 h at 37 °C. After the selected time of treatment, the luminescence signal was recorded with the multimodal plate reader GloMax Discover System (Promega GmbH, Walldorf, Germany).

### 4.11. Measurement of Changes in Intracellular Ca^2+^ Level

Changes in the intracellular Ca^2+^ level were measured by the fluorometric dye Fluo-4 AM. In brief, LS180 and SW620 cells were seeded as previously described for ROS and GSH levels. A total of 30 min before the end of treatment, 10 µM of the calcium ionophore ionomycin was added to the untreated cells as a positive control. After treatment, the culture medium was removed, cells were washed in prewarmed PBS, and 50 µL of Fluo-4 AM (5 µM in PBS) was added to each well and incubated for 2 h at 37 °C in the dark. Then plates were removed from the incubator and allowed to equilibrate to RT (30 min). After incubation, the dye was removed, and wells were washed in PBS. 100 µL of PBS was added to each well, and the fluorescent intensity was recorded at λ = 475 nm for excitation and at λ = 500–550 nm for emission on the GloMax Discover System. The intracellular Ca^2+^ level was expressed as the percentage of control (100%).

### 4.12. Measurement of Changes in ΔΨm

The loss of mitochondrial membrane potential (ΔΨm) was monitored by measuring the uptake of a mitochondria-specific dye, JC-1. The reflection of the mitochondrial polarization state is the JC-1 red to green fluorescence ratio—a decrease in this fluorescence ratio indicates a decrease in (ΔΨm) [64]. In brief, LS180 and SW620 cells were seeded as previously described but into black 96-well culture plates. After treatment, the culture medium was removed, cells were washed with prewarmed PBS, and then incubated with 100 µL/well of JC-1 (2 μM in PBS) at 37 °C for 30 min in the dark. After washing with PBS, the cells were resuspended in 100 µL/well PBS, and the fluorescence intensity was recorded at λ = 475 nm for excitation, at λ = 500–550 nm for emission of green fluorescence, at λ = 520 nm for excitation, and at λ = 580–640 nm for red fluorescence. The red-to-green fluorescence ratio was calculated.

### 4.13. ATP Assay

Cell cytotoxicity was determined based on the level of intracellular adenosine 5′-triphosphate (ATP). The ATP level was monitored using the CellTiter-Glo 2.0 Cell Viability Assay following the manufacturer’s instructions. In brief, LS180 and SW620 cells were seeded and treated as previously described, except that they were seeded into white 96-well culture plates. After 12, 24, and 48 h of treatment, luminescence signals were recorded with the GloMax Discover System. Intracellular ATP level was expressed as the percentage of control (100%).

### 4.14. Determination of Apoptosis

Caspases activity was measured using the Caspase-Glo^®^ 3/7 assay, Caspase-Glo^®^8 assay, and the Caspase-Glo^®^9 assay, following the manufacturer’s instructions, respectively. In brief, LS180 and SW620 cells were seeded and treated as previously described. Cells were then treated with liposomal berberine and free berberine in a dose-dependent manner and incubated for the selected time points—24 and 48 h at 37 °C. After 24 and 48 h of treatment, a luminescence signal was recorded with the GloMax Discover System. Caspase activity was expressed as the percentage of control (100%).

The detection of apoptosis was monitored in a real-time manner using the RealTime Glo Annexin V Apoptosis Assay following the manufacturer’s instructions. In brief, SW620 cells were seeded in 50 µL of growth medium in a white 96-well plate at a concentration of 1.5 × 10^4^ cells/well. A set of control wells (growth medium only) to determine background luminescence and background fluorescence were included. The appropriate dilution of berberine and its liposomal formulation were prepared in growth medium at 4× the desired final concentration. A set of control wells (untreated controls) were included. 50 µL of the free berberine (or its liposomal formulation) dilution was added to the appropriate wells in the 96-well plate. Then, 100 µL of 2× concentrated RealTime-Glo Annexin V Apoptosis and Necrosis Detection Reagent in growth medium was added to each well. Cells were incubated for 48 h in a humidified atmosphere containing 95% air and 5% CO_2_ at 37 °C. Luminescence and fluorescence (Ex 475, Em 500–550) were measured on the GloMax Discover System using the RealTime Glo Annexin V Apoptosis Assay pre-programmed protocol at the selected time intervals of 2, 4, 6, 8, 12, 14, 16, 18, 24, 26, 28, 30, 36, and 48 h. Apoptosis was expressed as the percentage of control (100%).

### 4.15. Determination of ICD Induction

#### 4.15.1. Surface Detection of CRT

LS180 and SW620 cells were seeded on cover glasses inside 24-well plates with 1 × 10^5^ cells/well and allowed to adhere overnight. Then cells were treated with berberine or its liposomal formulation (10 or 50 μM) for 24 h. After treatment, cells were placed on ice, washed twice with cold PBS, and fixed in 0.25% PFA in PBS for 5 min. Cells were then washed twice in PBS, and primary antibody against CRT diluted in cold blocking buffer (2% FBS w PBS) (1:200 dilution) was added for 30 min on ice at 4 °C. After three washes in cold PBS, cells were incubated for 30 min with Alexa Fluor 647-conjugated secondary antibody diluted in cold blocking buffer (1:200 dilution) on ice at 4 °C in the dark. Cells were washed with PBS and mounted on slides with the mounting medium, including DAPI. Cells were visualized with confocal microscopy (Leica SP8 LSM, Wetzlar, Germany). Images were taken using a 63× objective lens and processed using ImageJ-win64 software. The same procedure of macrophage treatment and staining was performed as described in the previous experiment.

#### 4.15.2. Intracellular Staining of CRT

The same procedure of cell seeding and treatment was performed as described in the previous experiment. After treatment, cells were washed with PBS, fixed with 4% PFA for 20 min at RT, permeabilized with 0.1% Triton X-100 for 10 min at RT, rinsed three times with PBS, and nonspecific binding sites were blocked with 10% FBS in PBS for 30 min at RT. Primary antibody against CRT diluted in cold blocking buffer (1:200 dilution) was added for 1 h. Subsequently, cells were washed three times with PBS and incubated for 30 min with Alexa Fluor 647-conjugated secondary antibody diluted in cold blocking buffer (1:200 dilution) at RT. Cells were washed with PBS and mounted on slides with the mounting medium, including DAPI. Cells were visualized with confocal microscopy. Images were taken using a 63× objective lens and processed using ImageJ software.

#### 4.15.3. RealTime-Glo™ Extracellular ATP Assay

Extracellular ATP release was monitored in a real-time manner using the RealTime-Glo Extracellular ATP Assay following the manufacturer’s instructions. In brief, SW620 cells were seeded in 100 µL of growth medium in a white 96-well plate at a concentration of 1.5 × 10^4^ cells/well. A set of control wells (growth medium only) to determine background luminescence were included. The appropriate dilution of berberine and its liposomal formulation were prepared in growth medium at 4× the desired final concentration. A set of control wells (untreated controls) were included. 50 µL of the free berberine (or its liposomal formulation) dilution was added to the appropriate wells in the 96-well plate. Then, 50 µL of 4× concentrated RealTime-Glo™ Extracellular ATP Assay Reagent in growth medium was added to each well. Cells were incubated for 24 h in a humidified atmosphere containing 95% air and 5% CO_2_ at 37 °C. Luminescence was measured on the GloMax Discover System using the RealTime-Glo™ Extracellular ATP Assay pre-programmed protocol at the selected time intervals of 2, 4, 6, 8, 14, 16, 18, 20, 22, and 24 h. The ATP level was expressed as the percentage of control (100%).

#### 4.15.4. Lumit HMGB1 Human Immunoassay

Extracellular HMGB1 release was measured using the Lumit HMGB1 Human Immunoassay following the manufacturer’s instructions *. In brief, SW620 cells were seeded in 50 µL of growth medium in transparent 96-well plates at a concentration of 1.5 × 10^4^ cells/well. A set of control wells (growth medium only) to determine background luminescence were included. The appropriate dilution of berberine and its liposomal formulation were prepared in growth medium at 2× the desired final concentration. A set of control wells (untreated controls) were included. A total of 50 µL of berberine (or its liposomal formulation) dilution (10, 50, 100, and 200 μM) was added to the appropriate wells in the 96-well plate. After 24 h of treatment, 50μL of cell medium from each well was transferred to the corresponding wells of a separate solid white (or white with a clear bottom) 96-well plate. Then, 50 µL of 2× antibody mixture (1:125 dilution of Lumit Anti-HMGB1 mAb-SmBiT and 1:125 dilution of Lumit Anti-hMGB1 mAb-LgBiT) was added. The plate was briefly mixed with a plate shaker (10 s at 500–750 rpm) and incubated at RT for 60–90 min. Following incubation, 25μL of Lumit Detection Reagent B were added, and luminescence was measured on the multimodal plate reader GloMax Discover System. Extracellular HMGB1 level was expressed as the percentage of control (100%). *(This was Promega prototype material from August 2021).

### 4.16. Determination of Phagocytosis

#### 4.16.1. Confocal-Based Phagocytosis Assay for Stimulated Macrophages

Differentiation into THP-1 macrophages was achieved by incubation of THP-1 monocytes seeded on cover glasses inside 12-well plates at 2 × 10^5^ cells/well in complete RPMI 1640 medium in the presence of 100 ng/mL (162 nM) PMA for 72 h. On the same day, LS180 and SW620 cells were plated in the complete growth medium in Petri dishes such that 80% confluency would be achieved 96 h later. After 72 h of THP-1 differentiation, the culture medium was removed and replaced with medium containing either berberine or its liposomal formulation at 10 or 50 μM for 24 h. For detection of phagocytosis, non-treated cancer cells were labeled with 5 μM CellTrace CFSE according to the manufacturer’s instructions. Then cancer cells were harvested using trypsin-EDTA and counted. Exactly 1 × 10^6^ cells/well were transferred to the wells with labeled THP-1 macrophages (1 μM CellTrace Far-Red) for 2 h at 37 °C. The final ratio of effector (THP-1) to target (cancer cells) was approximately 1:5. After 2 h of co-incubation of cancer cells with THP-1 macrophages, wells were rinsed with PBS, and the cells were fixed using 4% paraformaldehyde for 20 min at RT, washed in PBS, and mounted on slides with DAKO Mounting Medium. The cells were visualized with confocal microscopy. Images were taken using a 63× objective lens and processed using ImageJ software. Those macrophages in which green cells were ingested or partially ingested, hence showing green staining, were considered successfully phagocytic, while macrophages without green staining within the cells were not considered phagocytic.

#### 4.16.2. Confocal-Based Phagocytosis Assay for Treated Cancer Cells

The same procedure for THP-1 differentiation was performed as described in the previous experiment. In the meantime, after 48 h of THP-1 differentiation, LS180 and SW620 cells were plated onto Petri dishes in complete growth medium at a density of 3 × 10^6^ cells/dish and incubated overnight. The next day, cancer cells were treated with either berberine or its liposomal formulation (10 and 50 µM) for 24 h. In the meantime, after 72 h of THP-1 differentiation, the culture medium was removed and replaced with fresh, complete growth medium. The same procedure of cell treatment and staining was performed as described in the previous experiment.

#### 4.16.3. Fluorescence Plate Reader Based Phagocytosis Assay for Stimulated Macrophages

Alternatively, a fluorescence plate reader was used to assess phagocytosis. Differentiation into THP-1 macrophages was achieved by incubation of THP-1 monocytes seeded in black 96-well plates at a density of 2 × 10^4^/well in complete RPMI 1640 medium in the presence of 100 ng/mL PMA for 72 h at 37 °C. On the same day, LS180 and SW620 cells were plated in the complete growth medium in Petri dishes such that 80% confluency would be achieved 96 h later. After 72 h of THP-1 differentiation, the culture medium was removed and replaced with medium containing either berberine or its liposomal formulation at 0.2–50 μM for 24 h. In those experiments, only target cells were green labelled—labelled cancer cells were harvested by using a Trypsin-EDTA solution, counted, and added to the wells containing unstained THP-1 macrophages. The final ratio of effector (THP-1) to target (cancer cells) was approximately 1:5. The prepared plate was centrifuged for 5 min at 550× *g* at RT, then placed in a 5% CO_2_ and 37 °C incubator for 2 h at 37 °C. Additionally, some wells were treated with 10 µg/mL anti-CD47 antibody or with 20 µM ATP solution. After 2 h of co-incubation, cancer cells with THP-1 macrophages were washed to remove cells that were not phagocytized or “stuck” to phagocytes, followed by reading fluorescence intensities that reflected cells captured by macrophages. The fluorescence signal was recorded with the GloMax Discover System at λ = 475 nm for excitation and at λ = 500–550 nm for emission. All experiments were performed a minimum of three times in triplicate. The phagocytosis index, expressed as RFU, was calculated as follows:

(5) $$\%\;phagocytosis=\frac{(treated\;macrophages+untreated\;cells)-untreated\;macrophages}{(untreated\;macrophages+untreated\;cells)-untreated\;macrophages}\times 100\%$$

#### 4.16.4. Fluorescence Plate Reader-Based Phagocytosis Assay for Treated Cancer Cells

The same procedure for THP-1 differentiation was performed as described in the previous experiment. In the meantime, after 48 h of THP-1 differentiation, LS180 and SW620 cells were seeded onto 6-well plates in complete growth medium at a density of 0.5 × 10^6^/well and incubated overnight. The next day, cancer cells were treated with either berberine or its liposomal formulation (10 and 50 µM) for 24 h. In the meantime, after 72 h of THP-1 differentiation, the culture medium was removed and replaced with fresh, complete growth medium. The same procedure of cell treatment and staining was performed as described in the previous experiment. The same procedure of cell treatment and staining was performed as described in the previous experiment.

(6) $$\%\;phagocytosis=\frac{(untreated\;macrophages+treated\;cells)-untreated\;macrophages}{(untreated\;macrophages+untreated\;cells)-untreated\;macrophages}\times 100\%$$

## 5. Conclusions

Summarizing the presented data, the proposed mechanism of action of the studied liposomal berberine against colon cancer cell lines shared previously described principal biochemical features of CD47-mediated cell death, which are caspase independence, loss of Δψm, and leakage of Ca^2+^ ions [17]. At the same time, the above-mentioned features as well as intracellular ATP depletion due to oxidative stress induction (ROS generation, GSH depletion) are hallmarks of oncosis-like cell death.

In conclusion, this study demonstrated a novel anti-tumor mechanism of liposomal berberine and provided insights into CD47-targeted immunotherapy in colorectal cancer. We described the bi-directional action of berberine: antitumor activity against tumor cells and a macrophage-stimulating effect on the phagocytosis of tumor cells. Potential intravenous administration of berberine-loaded liposomes should result in both activation of immune cells and selective cytotoxicity on cancer cells to induce their cell death. This approach seems to have as much applicability as possible.

## Figures and Tables

**Table 1:** Characterization of liposomal formulations.

Liposomal Formulation	pH Gradient	Liposomes Size before Berberine Encapsulation [nm]	PDI	Liposomes Size after Berberine Encapsulation [nm]	PDI
HSPC:Chol:DSPE-PEG_2000_	Ammonium sulfate	117.1	0.036	118.2	0.049
	Citric acid	99.0	0.061	99.3	0.052
	Vitamin C	108.1	0.055	108.8	0.032

**Table 2:** Relative IC_50_ values [µM] of berberine, blank liposomes, and berberine-loaded liposomes were performed using the GraphPad Prism software (version 9, GraphPad Software, San Diego, CA, USA).

Formulation	IC_50_ (µM)							
	LS 180			SW620			CCD 112CoN	
	24 h	48 h	72 h	24 h	48 h	72 h	48 h	72 h
berberine	28 ± 6	6 ± 2	5 ± 2	56 ± 7	2 ± 1	2 ± 1	168 ± 29	53 ± 14
Lip ber vit C	178 ± 18	37 ± 7	11 ± 2	596 ± 83	25 ± 3	5 ± 1	205 ±5	144 ± 2
Lip vit C	124 ± 27	88 ± 6	51 ± 5	-	208	-	207 ± 41	72 ± 10
Lip ber CA	237 ± 69	123 ± 8	35 ± 3	-	-	42 ± 24	191 ± 32	153 ± 2
Lip CA	148 ± 8	112 ± 6	111 ± 2	-	-	2 ± 2	133 ± 18	136 ± 12
Lip ber AS	440 ± 40	103 ± 12	61 ± 21	355 ± 54	79 ± 32	18 ± 6	-	1109 ± 249
Lip AS		-	-	-	-	81 ± 3	-	-

## Data Availability

Data are contained within the article and the supplementary materials.

## Supplementary Materials

The following supporting information can be downloaded at: https://www.mdpi.com/article/10.3390/ph17010005/s1, Figure S1: LS180 viability after 24 h of berberine treatment; Figure S2: LS180 viability after 48 h of berberine treatment; Figure S3: LS180 viability after 72 h of berberine treatment; Figure S4: SW620 viability after 24 h of berberine treatment; Figure S5: SW620 viability after 48 h of berberine treatment; Figure S6: SW620 viability after 72 h of berberine treatment; Figure S7: CCD112CoN viability after 48 h of berberine treatment; Figure S8: CCD112CoN viability after 72 h of berberine treatment; Figure S9: Caspase 3/7 activity 24 h; Figure S10: Caspase 3/7 activity 48 h; Figure S11: Caspase 8 activity 24 h; Figure S12: Caspase 8 activity 48 h; Figure S13: Caspase 9 activity 24 h; Figure S14: Caspase 9 activity 48 h; Figure S15: Δψm changes 48 h; Figure S16: Intracellular Ca^2+^ level 48 h; Figure S17: ROS level 48 h; Figure S18: GSH level 48 h; Figure S19: Macrophages CRT cell surface staining after berberine pre-treatment; Table S1: Characterization of liposomal formulations after long-term storage.
