# Supplementary material for: Dual Role of Vitamin C-Encapsulated Liposomal Berberine in Effective Colon Anticancer Immunotherapy
Source: Pharmaceuticals (Basel). 2023 Dec 20;17(1):5. doi: 10.3390/ph17010005 (PMC10819181; doi:10.3390/ph17010005)
Supplement: Supplementary file 1 [file pharmaceuticals-17-00005-s001.zip › pharmaceuticals-2737961-supplementary.pdf]

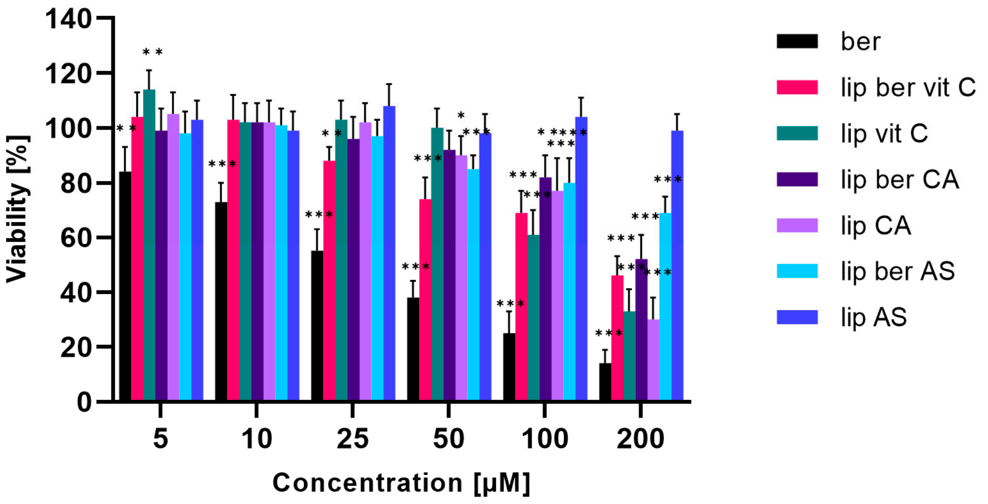

Figure S1. Cell viability of LS180 cell line after 24 hours administration of berberine-loaded liposomes, blank liposomes, and free berberine. Viability of untreated cells (control) was considered to be 100% of cell viability. Data represent the mean ± SD of three independent biological replicates. Statistical significance analysis of data differences was performed using the GraphPad Prism software (version 9, GraphPad Software, San Diego, CA, USA). Statistical significance was determined using a one-way ANOVA (Dunnett's modification) test. *** p <0.001; ** p < 0.002; * p <0.033.

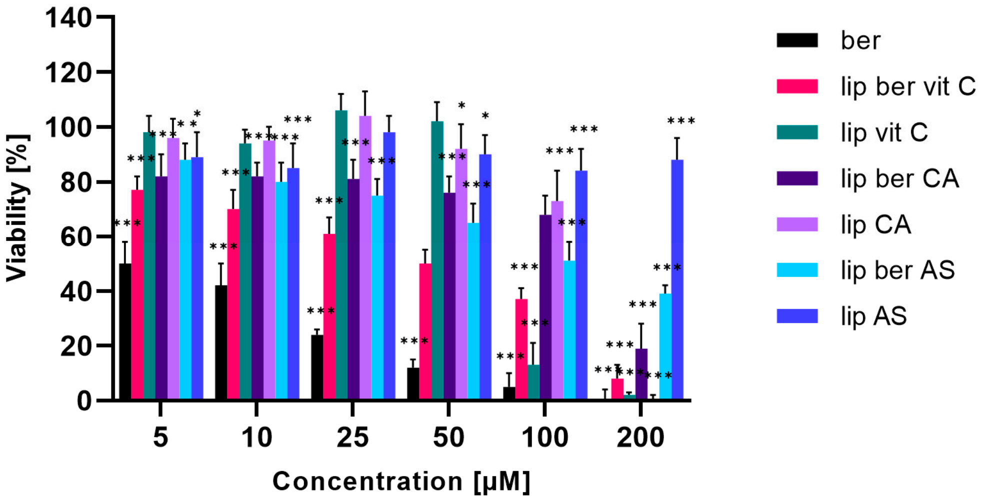

Figure S2. Cell viability of LS180 cell line after 48 hours administration of berberine-loaded liposomes, blank liposomes, and free berberine. Viability of untreated cells (control) was considered to be 100% of cell viability. Data represent the mean ± SD of three independent biological replicates. Statistical significance analysis of data differences was performed using the GraphPad Prism software (version 9, GraphPad Software, San Diego, CA, USA). Statistical significance was determined using a one-way ANOVA (Dunnett's modification) test. *** p <0.001; ** p < 0.002; * p <0.033.

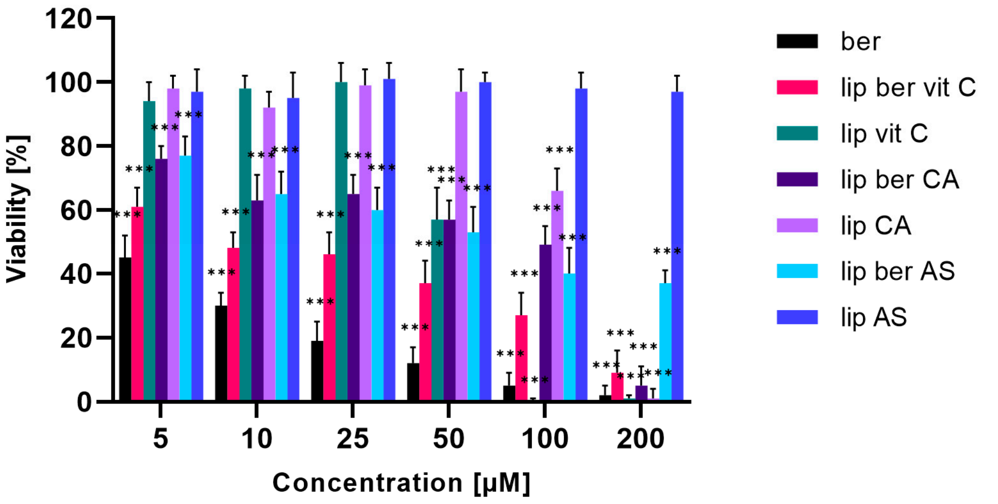

Figure S3. Cell viability of LS180 cell line after 72 hours administration of berberine-loaded liposomes, blank liposomes, and free berberine. Viability of untreated cells (control) was considered to be 100% of cell viability. Data represent the mean ± SD of three independent biological replicates. Statistical significance analysis of data differences was performed using the GraphPad Prism software (version 9, GraphPad Software, San Diego, CA, USA). Statistical significance was determined using a one-way ANOVA (Dunnett's modification) test. *** p <0.001; ** p < 0.002; * p <0.033.

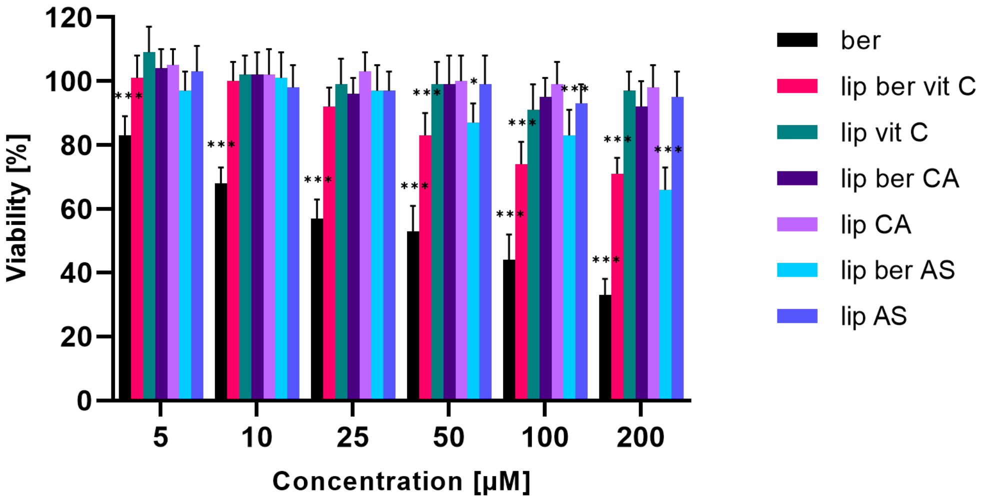

Figure S4. Cell viability of SW620 cell line after 24 hours administration of berberine-loaded liposomes, blank liposomes, and free berberine. Viability of untreated cells (control) was considered to be 100% of cell viability. Data represent the mean ± SD of three independent biological replicates. Statistical significance analysis of data differences was performed using the GraphPad Prism software (version 9, GraphPad Software, San Diego, CA, USA). Statistical significance was determined using a one-way ANOVA (Dunnett's modification) test. *** p <0.001; ** p < 0.002; * p <0.033.

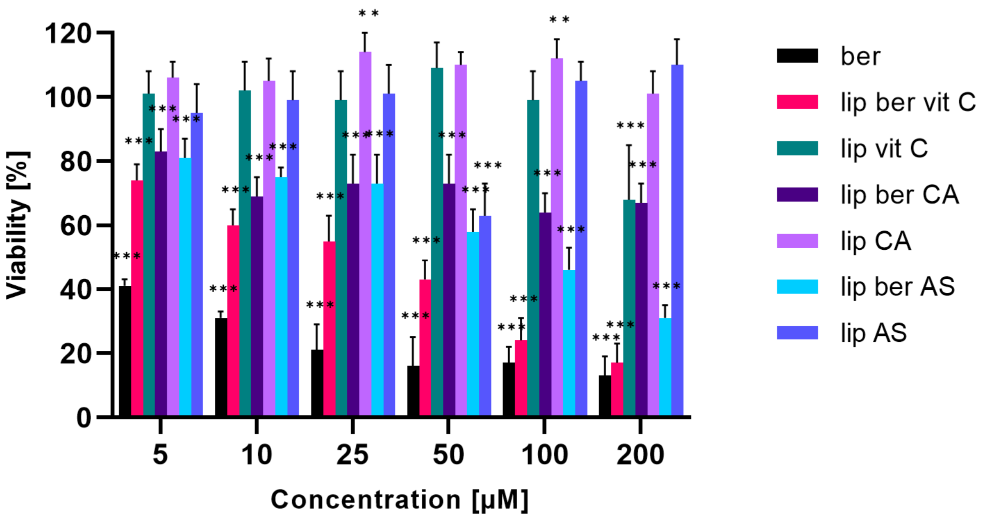

Figure S5. Cell viability of SW620 cell line after 48 hours administration of berberine-loaded liposomes, blank liposomes, and free berberine. Viability of untreated cells (control) was considered to be 100% of cell viability. Data represent the mean ± SD of three independent biological replicates. Statistical significance analysis of data differences was performed using the GraphPad Prism software (version 9, GraphPad Software, San Diego, CA, USA). Statistical significance was determined using a one-way ANOVA (Dunnett's modification) test. *** p <0.001; ** p < 0.002; * p <0.033.

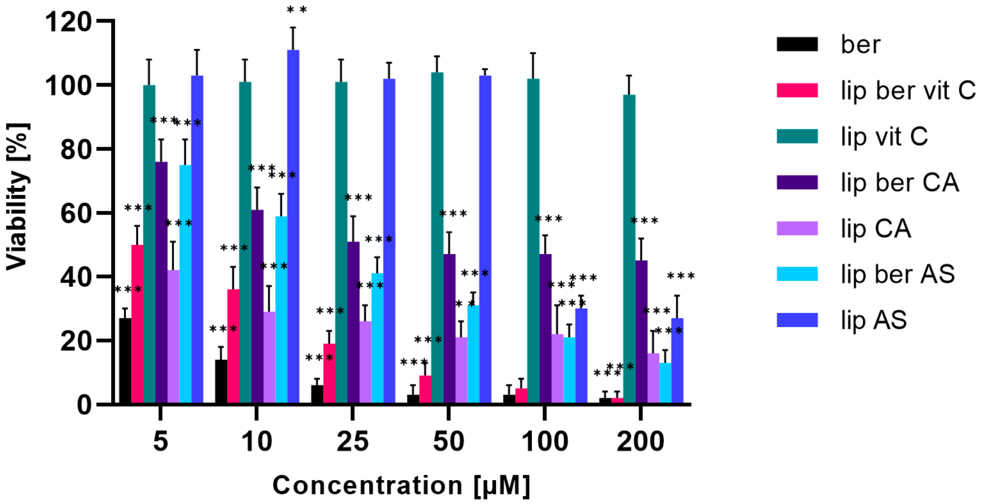

Figure S6. Cell viability of SW620 cell line after 72 hours administration of berberine-loaded liposomes, blank liposomes, and free berberine. Viability of untreated cells (control) was considered to be 100% of cell viability. Data represent the mean ± SD of three independent biological replicates. Statistical significance analysis of data differences was performed using the GraphPad Prism software (version 9, GraphPad Software, San Diego, CA, USA). Statistical significance was determined using a one-way ANOVA (Dunnett's modification) test. *** p <0.001; ** p < 0.002; * p <0.033.

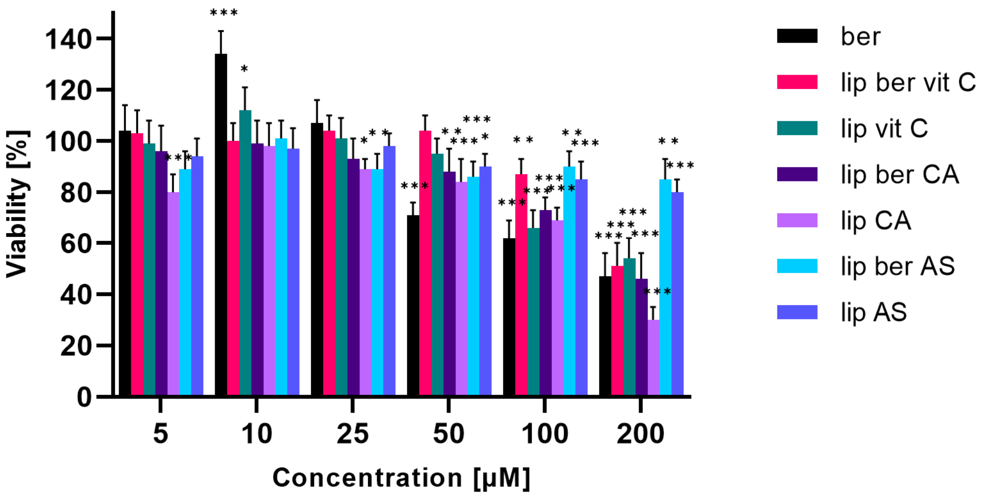

Figure S7. Cell viability of CCD112CoN cell line after 48 hours administration of berberine-loaded liposomes, blank liposomes, and free berberine. Viability of untreated cells (control) was considered to be 100% of cell viability. Data represent the mean ± SD of three independent biological replicates. Statistical significance analysis of data differences was performed using the GraphPad Prism software (version 9, GraphPad Software, San Diego, CA, USA). Statistical significance was determined using a one-way ANOVA (Dunnett's modification) test. *** p <0.001; ** $p < 0.002$; * p <0.033.

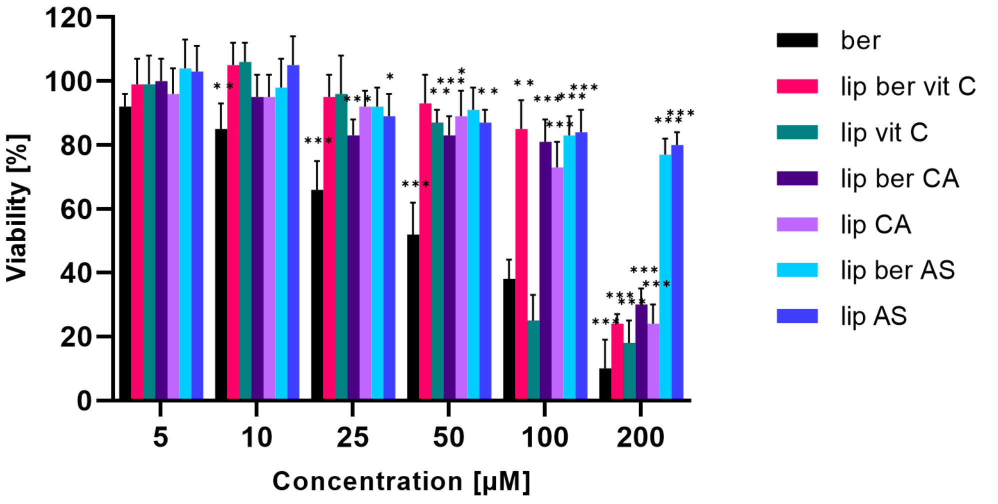

Figure S8. Cell viability of CCD112CoN cell line after 72 hours administration of berberine-loaded liposomes, blank liposomes, and free berberine. Viability of untreated cells (control) was considered to be 100% of cell viability. Data represent the mean ± SD of three independent biological replicates. Statistical significance analysis of data differences was performed using the GraphPad Prism software (version 9, GraphPad Software, San Diego, CA, USA). Statistical significance was determined using a one-way ANOVA (Dunnett's modification) test. *** p <0.001; ** $p < 0.002$; * p <0.033.

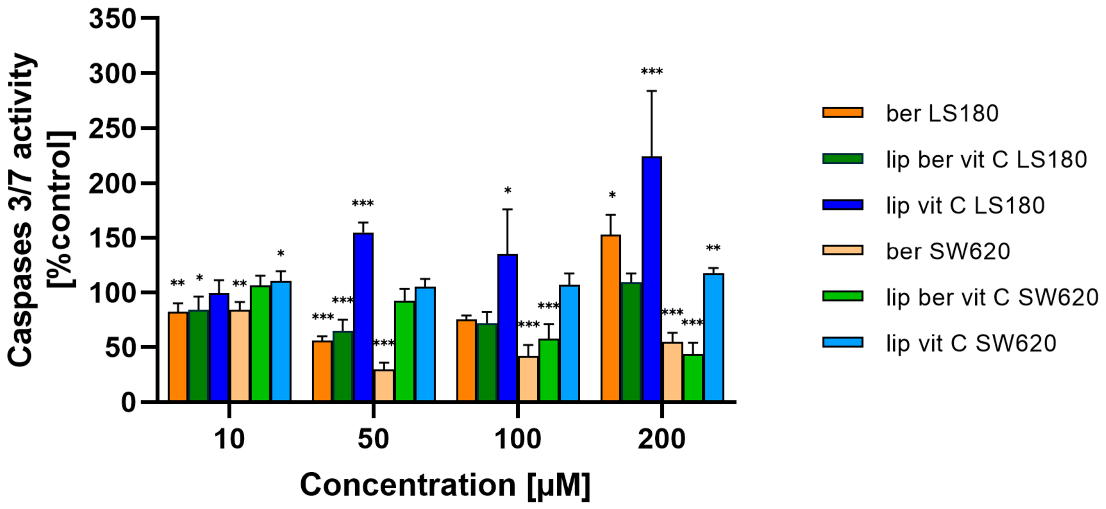

Figure S9. Caspases 3/7 changes in colon cancer cells after treatment of berberine loaded liposomes, blank-liposomes, and free berberine (10, 50, 100, or 200 µM) for 24 h. Luminescence of untreated cells (control) was considered to be 100% of the caspases 3/7 level. Data represent the mean ± SD of three independent biological replicates. Statistical significance was determined using a one-way ANOVA (Dunnett's modification) test. *** p <0.001; ** p < 0.002; * p <0.033.

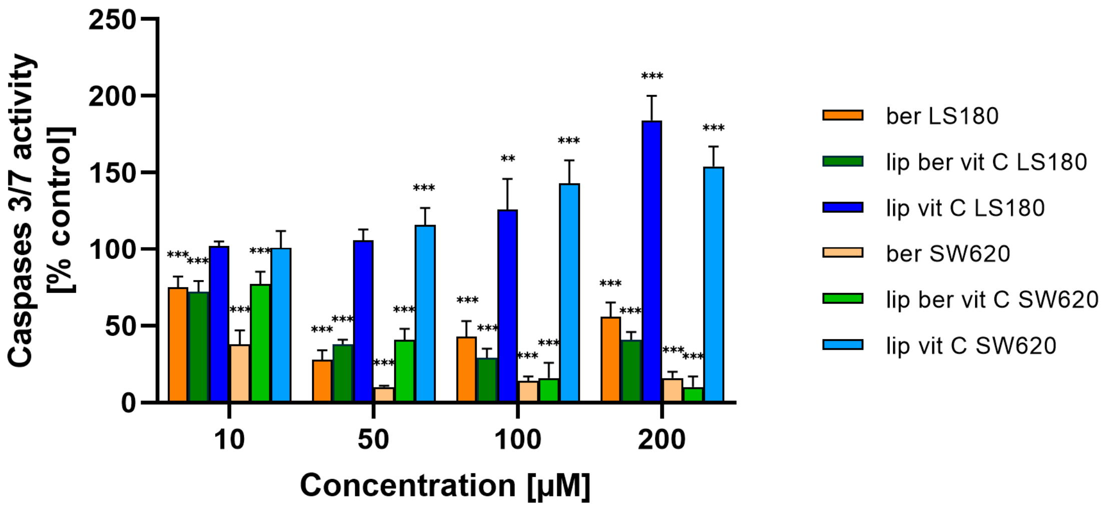

Figure S10. Caspases 3/7 changes in colon cancer cells after treatment of berberine loaded liposomes, blank-liposomes, and free berberine (10, 50, 100, or 200 µM) for 48 h. Luminescence of untreated cells (control) was considered to be 100% of the caspases 3/7 level. Data represent the mean ± SD of three independent biological replicates. Statistical significance was determined using a one-way ANOVA (Dunnett's modification) test. *** p <0.001; ** p < 0.002; * p <0.033.

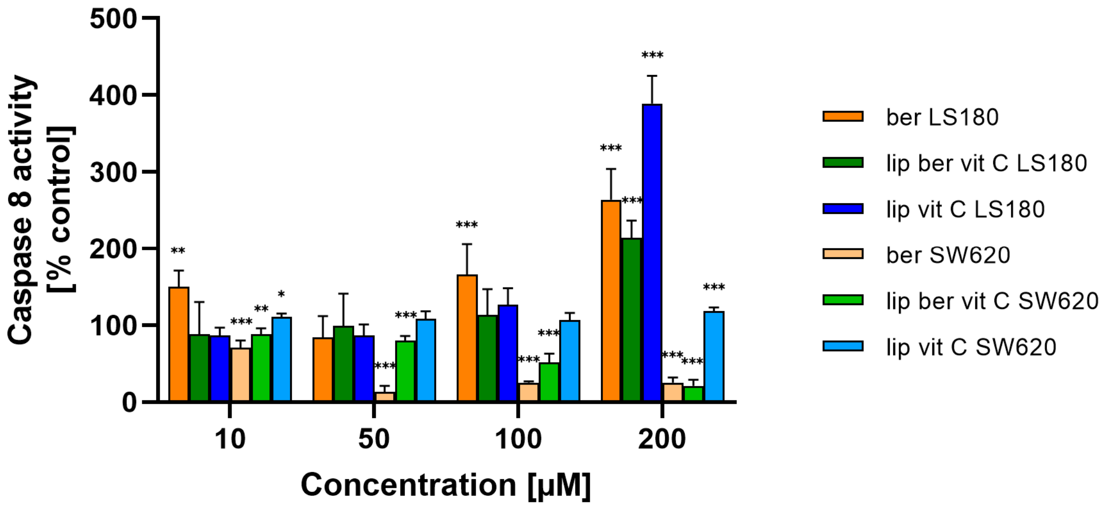

Figure S11. Caspase 8 changes in colon cancer cells after treatment of berberine loaded liposomes, blank-liposomes, and free berberine (10, 50, 100, or 200 µM) for 24 h. Luminescence of untreated cells (control) was considered to be 100% of the caspase 8 level. Data represent the mean ± SD of three independent biological replicates. Statistical significance was determined using a one-way ANOVA (Dunnett's modification) test. *** p <0.001; ** p < 0.002; * p <0.033.

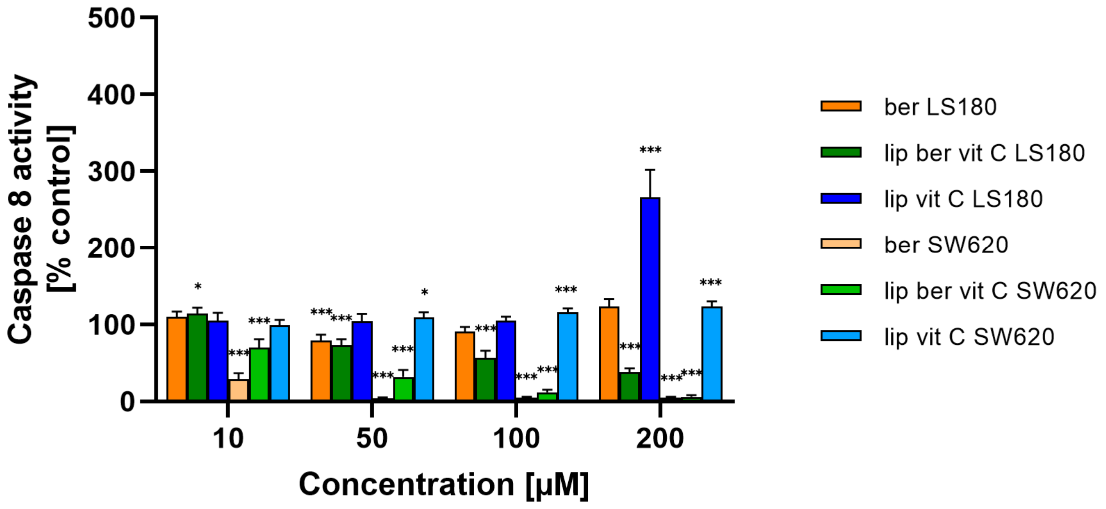

Figure S12. Caspase 8 changes in colon cancer cells after treatment of berberine loaded liposomes, blank-liposomes, and free berberine (10, 50, 100, or 200 µM) for 48 h. Luminescence of untreated cells (control) was considered to be 100% of the caspase 8 level. Data represent the mean ± SD of three independent biological replicates. Statistical significance was determined using a one-way ANOVA (Dunnett's modification) test. *** p <0.001; ** p < 0.002; * p <0.033.

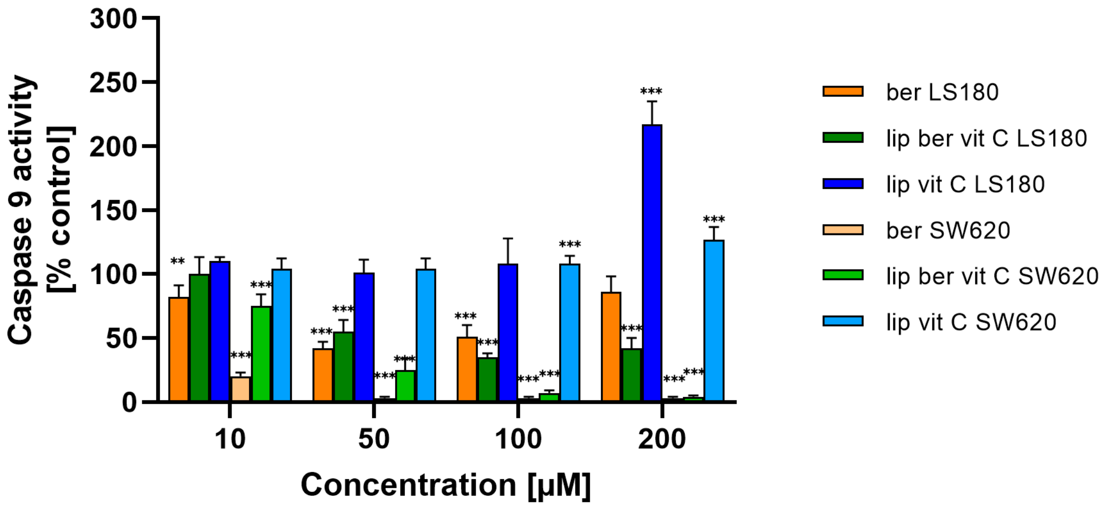

Figure S13. Caspase 9 changes in colon cancer cells after treatment of berberine loaded liposomes, blank-liposomes, and free berberine (10, 50, 100, or 200 μM) for 24 h. Luminescence of untreated cells (control) was considered to be 100% of the caspase 9 level. Data represent the mean ± SD of three independent biological replicates. Statistical significance was determined using a one-way ANOVA (Dunnett's modification) test. *** p <0.001; ** p < 0.002; * p <0.033.

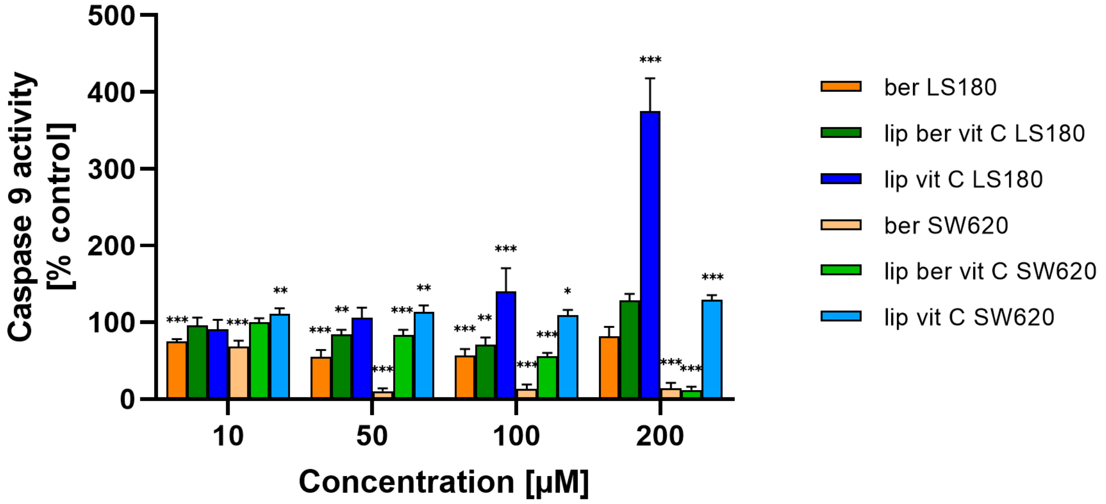

Figure S14. Caspase 9 changes in colon cancer cells after treatment of berberine loaded liposomes, blank-liposomes, and free berberine (10, 50, 100, or 200 μM) for 48 h. Luminescence of untreated cells (control) was considered to be 100% of the caspase 9 level. Data represent the mean ± SD of three independent biological replicates. Statistical significance was determined using a one-way ANOVA (Dunnett's modification) test. *** p <0.001; ** p < 0.002; * p <0.033.

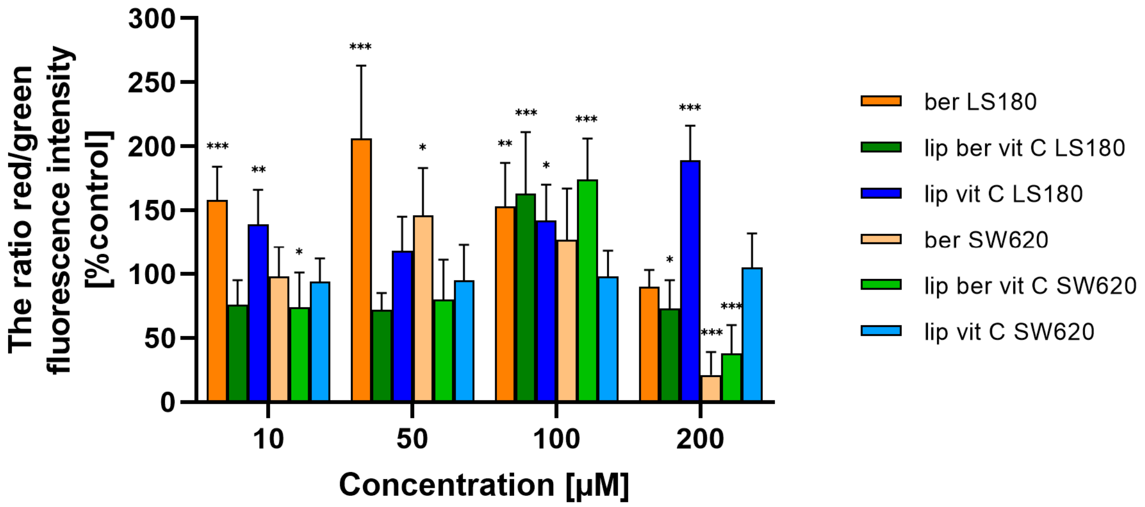

Figure S15. Δψm changes in colon cancer cells after treatment of berberine loaded liposomes, blank-liposomes, and free berberine (10, 50, 100, or 200 μM) for 48 h. Δψm was determined by fluorometry. The ratio of red to green fluorescence intensity of untreated cells (control) was considered to be 100% of the Δψm level. Data represent the mean ± SD of three independent biological replicates. Statistical significance was determined using a one-way ANOVA (Dunnett's modification) test. *** p <0.001; ** p < 0.002; * p <0.033.

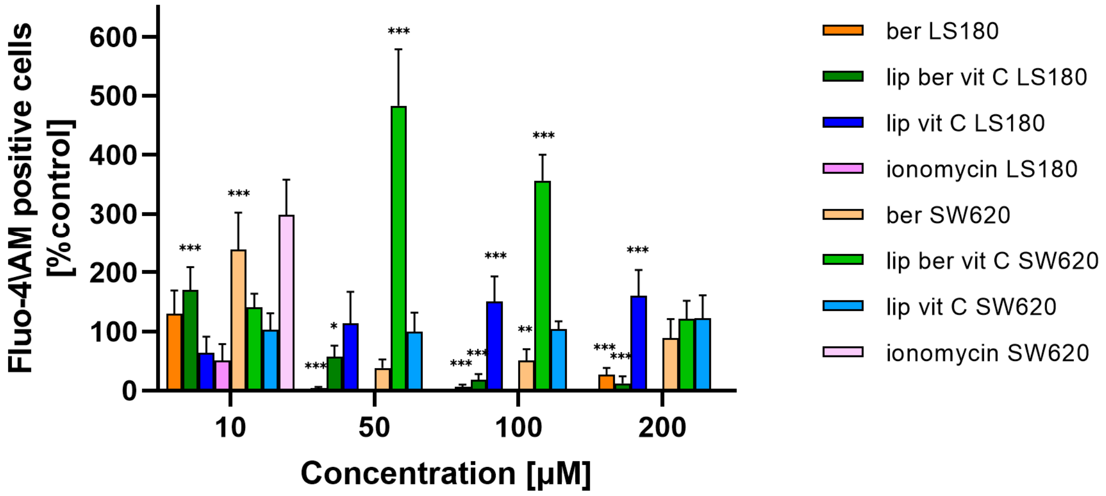

Figure S16. Changes in Ca^{2+} level in colon cancer cells after treatment with free berberine, blank liposomes, or berberine-loaded liposomes (10, 50, 100, or 200 μM) for 48 h. RFU of untreated cells (control) was considered to be 100% of the Ca^{2+} level. Data represent the mean ± SD of four independent biological replicates. Statistical significance was determined using a one-way ANOVA (Dunnett's modification) test. *** p <0.001; ** p < 0.002; * p <0.033.

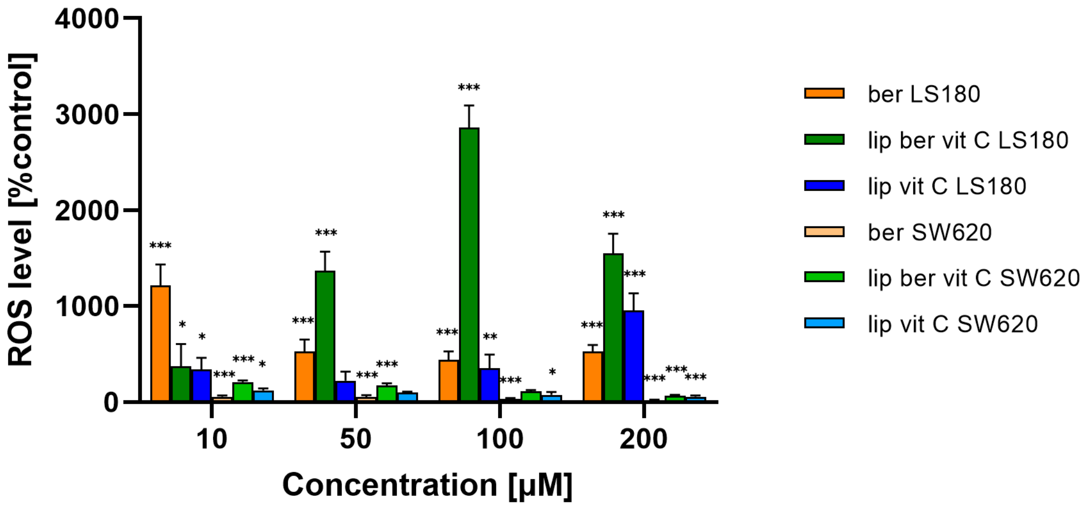

Figure S17. Increased ROS level in colon cancer cells after treatment with free berberine, blank liposomes, or berberine-loaded liposomes (10, 50, 100, or 200 μM) for 48 h. Luminescence of untreated cells (control) was considered to be 100% of the ROS level. Data represent the mean ± SD of two independent biological replicates. Statistical significance was determined using a one-way ANOVA (Dunnett's modification) test. *** $p < 0.001$; ** $p < 0.002$; * $p < 0.033$.

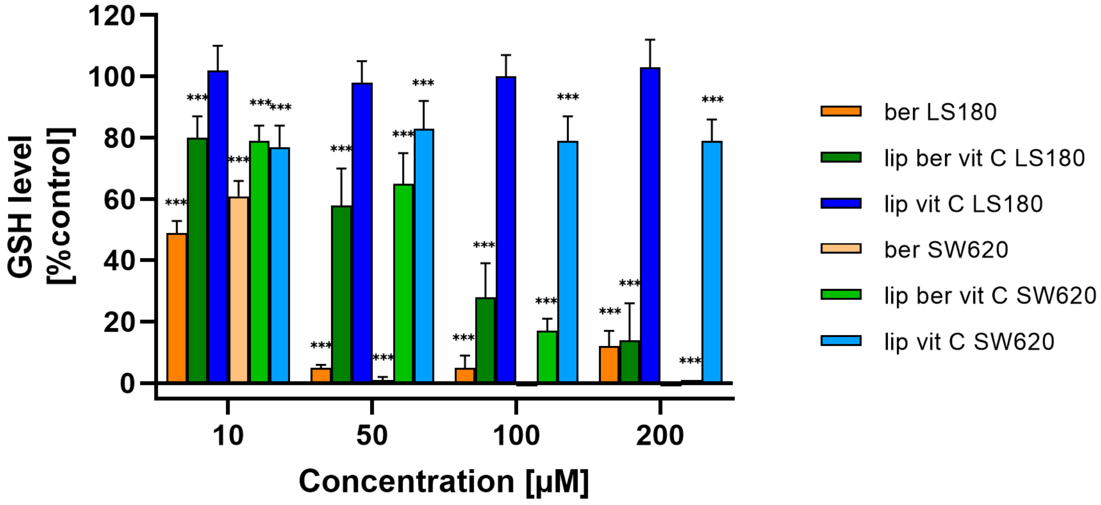

Figure S18. Decreased GSH level in colon cancer cells after treatment with free berberine, blank liposomes, or berberine-loaded liposomes (10, 50, 100, or 200 μM) for 48 h. Luminescence of untreated cells (control) is considered to be 100% of the GSH level. Data represent the mean ± SD of three independent replicates. Statistical significance was determined using a one-way ANOVA (Dunnett's modification) test. *** $p < 0.001$; ** $p < 0.002$; * $p < 0.033$.

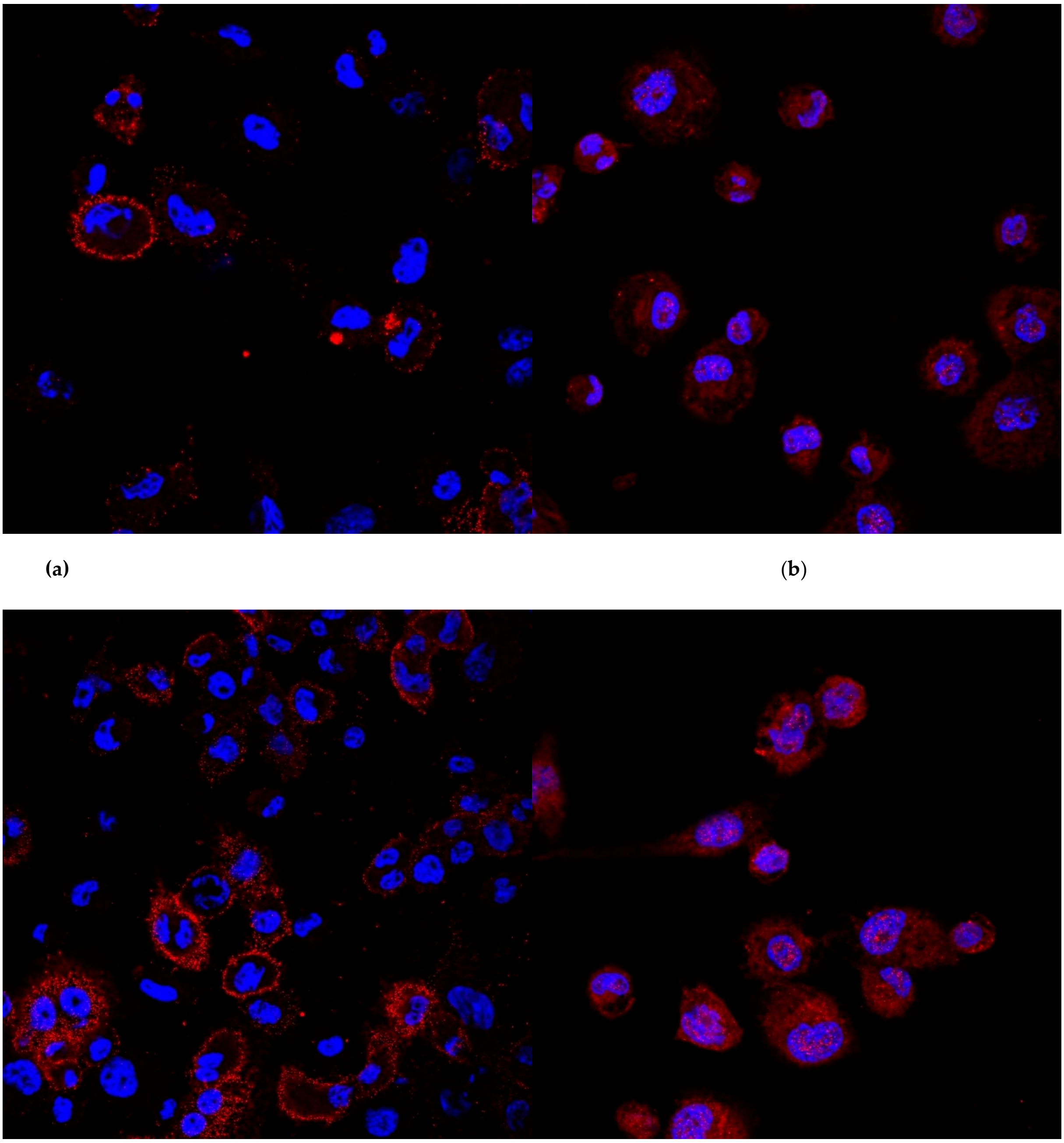

(a) (b)

(c) (d)

Figure S19. Microscopic photo of THP-1 cells (**a**) after treatment with 50μM berberine-loaded liposomes with vitamin C 10μM berberine – surface staining (**b**) after treatment with 50μM berberine-loaded liposomes with vitamin C – intracellular staining (**c**) after treatment with 10μM berberine – surface staining (**d**) after treatment with 10μM berberine – intracellular staining. The cell nuclei were stained with DAPI and cell surface CRT bound by anti-CRT antibody recognized by Alexa Fluor 647 conjugated secondary antibody.

Table S1. Characterization of liposomal formulations during long term storage.

Time	Size [nm]	SD	Size [nm]	SD	Size [nm]	SD
days	Lip ber vit C		Lip ber CA		Lip ber AS	
**1**	112	1,65	110	0,64	125	0,93
**20**	109	0,49	110	0,87	121	0,61
**40**	108	0,98	108	0,44	119	1,08
**60**	108	0,40	108	1,07	121	1,57
**80**	108	0,67	107	0,65	123	1,15
**365**	108	1,25	109	4,17	125	2,20

Time	PDI	SD	PDI	SD	PDI	SD
days	Lip ber vit C		Lip ber CA		Lip ber AS	
**1**	0,063	0,015	0,030	0,016	0,073	0,019
**20**	0,048	0,010	0,061	0,010	0,111	0,012
**40**	0,063	0,025	0,047	0,016	0,114	0,022
**60**	0,072	0,020	0,081	0,057	0,096	0,034
**80**	0,051	0,002	0,053	0,021	0,066	0,022
**365**	0,089	0,014	0,181	0,009	0,073	0,027
